# Author Correction: Revealing the mechanism for covalent inhibition of glycoside hydrolases by carbasugars at an atomic level

**DOI:** 10.1038/s41467-018-06264-4

**Published:** 2018-09-07

**Authors:** Weiwu Ren, Robert Pengelly, Marco Farren-Dai, Saeideh Shamsi Kazem Abadi, Verena Oehler, Oluwafemi Akintola, Jason Draper, Michael Meanwell, Saswati Chakladar, Katarzyna Świderek, Vicent Moliner, Robert Britton, Tracey M. Gloster, Andrew J. Bennet

**Affiliations:** 1 Department of Chemistry, Simon Fraser University, 8888 University Drive, Burnaby, BC V5A 1S6 Canada; 2 Biomedical Sciences Research Complex, University of St. Andrews, North Haugh, St. Andrews, Fife KY16 9ST UK; 3 Department of Molecular Biology and Biochemistry, Simon Fraser University, 8888 University Drive, Burnaby, BC V5A 1S6 Canada; 4 Department de Química Física i Analítica, Universitat Jaume I, 12071 Castellón, Spain

Correction to: *Nature Communications*; 10.1038/s41467-018-05702-7; published online: 13 Aug 2018

In the originally published version of this article, the affiliation details for Tracey M. Gloster were incorrectly given as 'Department of Molecular Biology and Biochemistry, Simon Fraser University, 8888 University Drive, Burnaby, BC V5A 1S6, Canada'. The correct affiliation is 'Biomedical Sciences Research Complex, University of St. Andrews, North Haugh, St. Andrews, Fife KY16 9ST, UK'. This has now been corrected in both the PDF and HTML versions of the Article.

