# Supplementary material for: Revealing the mechanism for covalent inhibition of glycoside hydrolases by carbasugars at an atomic level
Source: Nat Commun. 2018 Aug 13;9:3243. doi: 10.1038/s41467-018-05702-7 (PMC6089974; doi:10.1038/s41467-018-05702-7)
Supplement: Supplementary file 1 — Supplementary Information [file 41467_2018_5702_MOESM1_ESM.pdf]

Supplementary Information for

Revealing the mechanism for covalent inhibition of glycoside hydrolases by carbasugars at an atomic level

Ren et al.

Supplementary Methods

All anhydrous reactions described were performed under an atmosphere of nitrogen using flamedried glassware. Normal phase column chromatography was carried out with 230-400 mesh silica gel (Silicycle, SiliaFlash® P60). Concentration and removal of trace solvents was done with a Büchi rotary evaporator using a dry ice/acetone condenser and vacuum applied from a Büchi V-500 pump. All reagents and starting materials were purchased from Sigma Aldrich, Alfa Aesar, TCI America or Arcos and were used without further purification. All solvents were purchased from Sigma Aldrich, EMD, Anachemia, Caledon, Fisher or ACP and used without further purification unless otherwise specified. CH_2Cl_2 was freshly distilled over CaH_2; Tetrahydrofuran (THF) was freshly distilled over Na metal/benzophenone. Cold temperatures were maintained by use of the following conditions: 0 °C, ice-water bath; −78 °C, acetone-dry ice bath; temperatures between −78 °C and 0 °C required for longer reaction times were maintained with a Neslab Cryocool Immersion Cooler (CC-100 II) in a 2-propanol bath.

Nuclear magnetic resonance (NMR) spectra were recorded on a Bruker Avance 600 equipped with a QNP or TCI cryoprobe (600 MHz), Bruker 500 (500 MHz), or Bruker 400 (400 MHz) using $CDCl_3$ or CD_3OD as solvent. Signal positions (δ) are given in parts per million from tetramethylsilane (δ 0) and were measured relative to the signal of the solvent (^{1}H NMR: $CDCl_3$: δ 7.26, CD_3OD: δ 3.31; ^{13}C NMR: $CDCl_3$: δ 77.16, CD_3OD: δ 49.00). Coupling constants (*J* values) are given in Hertz (Hz) and are reported to the nearest 0.1 Hz. ^{1}H NMR spectral data are tabulated in the order: multiplicity (s, singlet; d, doublet; t, triplet; q, quartet; m, multiplet; br., broad), coupling constants, number of protons. Infrared (IR) spectra were recorded on a Perkin Elmer Spectrum Two™ Fourier transform spectrometer with neat samples. Only selected characteristic absorption data are provided for each compound. High resolution mass spectra were performed on an Agilent 6210 TOF LC/MS using ESI-MS. Optical rotations were measured using a Perkin Elmer 341 Polarimeter at 589 nm.

Cyclophellitol **8** was synthesized by following a published procedure[1], and displayed identical physical properties to those reported.

(1*R*,2*S*,3*S*,6*S*)-4-(hydroxymethyl)-6-(3,5-difluorophenoxy)-cyclohex-4-ene-1,2,3-triol (3a) A suspension of NaH in oil (60%, 99 mg, 2.5 mmol) was washed with hexane (2 × 5 mL) before being transferred in dry DMSO (50 mL) to a 100 mL flask maintained at 18 °C. To this mixture a solution of (1*S*,4*S*,5*S*,6*S*)-4,5,6-tribenyzloxy-3-((benzyloxy)methyl)cyclohex-2-en-1-ol[2] (200 mg, 0.37 mmol) in dry DMSO (25 mL) was added dropwise. This mixture was left for 30 min at 18 °C before addition of potassium benzoate (100 mg, 0.62 mmol). After a further 30 min, 1,3,5-trifluorobenzene (700 μL, 6.7 mmol) was added slowly. After 30 min the reaction mixture a saturated NH_4Cl solution (20 mL) was added. Addition of brine (50 mL) was followed by extraction of the product from the aqueous solution with ether (3 × 50 mL). The combined organic layer was dried ($MgSO_4$), and concentrated under reduced pressure. The residue was purified by flash column chromatography (20% EtOAc/Hexane) to give a colorless syrup (170 mg, 70%) in >98% purity as determined by ^{1}H NMR spectroscopy. This material was used directly without further purification. To a solution of this 3,5-difluorophenyl ether (170 mg) in dry CH_2Cl_2 (50 mL) under an argon atmosphere, boron trichloride (5.0 equiv., 1 M soln in CH_2Cl_2) was added slowly *via* a syringe at –78 °C, and the mixture was maintained at this temperature whilst being stirred for 30 min. Subsequently the resultant mixture was allowed to warm up to 0 °C over a period of 30 min. When TLC analysis (20% EtOAc/Hexane) showed that the reaction was complete a solution of 1:1 MeOH–CH_2Cl_2 (5 mL) was added. The volatiles were removed under diminished pressure and the resultant residue was washed with CH_2Cl_2 (5 × 10 mL). Upon removal of solvent a white solid was obtained and recrystallized from MeOH gave the final compound **3a** (50 mg, 66 %); Mpt = 170–171 °C; $[\alpha]^{20}_D$ = +101.5° (6.9 mM in MeOH); ^{1}H NMR (500 MHz, CD_3OD) δ 6.55 (dd, J = 9.3, 2.0, 2H, H-2′, H-6′), 6.40 (m, 1H, H-4′), 5.90 (d, $J_{5,6}$ = 3.6, 1H, H-5), 5.01 (app.br s, 1H, H-6), 4.32 (d, 1H, $J_{3,2}$ = 4.0, H-3), 4.24-4.13 (m, 3H, H-2, H-7a, H-7b), 3.93 (dd, 1H, $J_{1,2}$ = 8.8, $J_{1,6}$ = 4.1, H-1), 3.26 (s, 1H, OH); ^{13}C NMR (151 MHz, CD_3OD) δ 165.13 (dd, $^1J_{C,F}$ = 244.8, $^3J_{C,F}$ = 16.0, C-3', C-5'), 162.14 (t, $^3J_{C,F}$ = 13.9, C-1′), 144.46 (C-4, Alkene), 120.18 (C-5, Alkene), 100.6 (m, C-2', C-6'), 97.00 (t, $^3J_{C,F}$ = 26.4, C-4′), 75.21 (C-6), 70.92 (C-1), 69.94 (C-2), 67.88 (C-3), 63.64 (C-7); analysis (calcd., found for $C_{13}H_{14}F_2O_5$): C (54.17, 54.10), H (4.90, 4.82).

(*R*)-3-((triisopropylsilyl)oxy)pent-4-enal (9): To a solution of (2*R*,3*R*)-2-iodomethyl-5-methoxytetrahydrofuran-3-ol[3] (5.00 g, 19.4 mmol) in DMF (32 mL) was added imidazole (2.90 g, 42.6 mmol), 4-dimethylaminopyridine (23 mg, 0.194 mmol), and TIPS-Cl (4.56 mL, 21.3 mmol). The mixture was stirred for 16 h and then was quenched with H_2O and the mixture was extracted with Et_2O. The combined organic layers were washed with brine and then were dried over Na_2SO_4. The solvents were removed *in vacuo* and the residue was then purified by flash column chromatography (CH_2Cl_2:pentane, 3:2) to yield the acetal as a colorless oil (6.42 g, 80%). To the above acetal in THF/H_2O (4/1, 70 mL) was added Zn dust (10.14 g, 15.5 mmol). The resulting cloudy suspension was refluxed for 2 h, cooled to room temperature, and filtered through a Celite® pad (diethyl ether rinse). The solution was further diluted with diethyl ether and was washed with brine and then dried over Na_2SO_4. The solvents were removed *in vacuo* to yield **9** as a colorless oil without further purification (3.97 g, 100%).

$[\alpha]_D^{20}$ = –13.9 (8.6 mM in $CHCl_3$); IR (neat): 2945, 2868, 1727, 1466, 1099 cm^{-1}; ^1H NMR (500 MHz, $CDCl_3$): δ 9.81 (t, J = 2.5 Hz, 1H), 5.92 (ddd, J = 17.1, 10.4, 6.1 Hz, 1H), 5.28 (apparent dt, J = 17.1, 1.2 Hz, 1H), 5.14 (apparent dt, J = 10.2, 1.2 Hz, 1H), 4.78-4.74 (m, 1H), 2.62 (dd, J = 5.6, 2.5 Hz, 2H), 1.08-1.04 (m, 21H); ^{13}C NMR (151 MHz, $CDCl_3$) δ 201.9, 140.3, 115.2, 70.0, 51.7, 18.17, 18.15, 12.4; HRMS (ESI): m/z $[M + H]^+$ calcd for $C_{14}H_{29}O_2Si$: 257.1931; found: 257.1928.

Ketone 13: To a solution of **9** (4.66 g, 18.2 mmol) in CH_2Cl_2 (90 mL) were added (*R*)-proline (1.62 g, 14.0 mmol), *N*-chlorosuccinimide (2.12 g, 15.9 mmol), and 2,2-dimethyl-1,3-dioxan-5-one (**12**, 2.20 mL, 18.7 mmol). The mixture was stirred at ambient temperature for 24 h and then was treated with H_2O. The mixture was extracted with Et_2O and the combined organic layers were washed with brine and then were dried over Na_2SO_4. The solvents were removed *in vacuo* and the residue was then purified by flash column chromatography (pentane:diethyl ether, 8:1) to yield **13** as a colorless oil (4.59 g, 60%).

$[\alpha]_D^{20}$ = +88.5 (21 mM in $CHCl_3$); IR (neat): 3538, 2944, 2868, 1738, 1223, 1086 cm^{-1}; ^1H NMR (600 MHz, $CDCl_3$) δ: 5.87 (ddd, *J* = 17.2, 10.3, 7.8 Hz, 1H), 5.34 (apparent dt, *J* = 17.2, 1.0 Hz,

1H), 5.28 (apparent dt, *J* = 10.3, 0.9 Hz, 1H), 4.57 (apparent t, *J* = 7.7 Hz, 1H), 4.46 (ddd, *J* = 8.9, 2.5, 1.5 Hz, 1H), 4.37 (dd, *J* = 8.9, 1.3 Hz, 1H), 4.29 (dd *J* = 17.6, 1.4 Hz, 1H), 4.08 (d, *J* = 17.6 Hz, 1H), 3.99 (dd, *J* = 7.5, 1.0 Hz, 1H), 3.48 (dd, *J* = 2.5, 0.9 Hz, 1H), 1.50 (s, 3H), 1.42 (s, 3H), 1.08-1.05 (m, 21H); ^{13}C NMR (151 MHz, $CDCl_3$) δ: 211.4, 138.4, 118.6, 101.7, 75.6, 72.8, 67.9, 66.6, 63.5, 24.0, 23.5, 18.15, 18.13, 12.6; HRMS (ESI): m/z $[M + H]^+$ calcd for $C_{20}H_{38}ClO_5Si$, 421.2172; found, 421.2188.

Alkene 14: To a cooled (–78 °C) solution of 5-(methanesulfonyl)-1-phenyl-1*H*-tetrazole (4.62 g, 20. 8 mmol) in THF (60 mL) was added dropwise LiHMDS (20.8 mL, 1.0 M in THF, 20.8 mmol) and stirred at –78 °C for 30 min. To this yellow solution **13** (4.40 g, 10.4 mmol) in THF (20 mL) was added dropwise at –78 °C and the reaction mixture was stirred for an additional 1 h before quenching with H_2O. The mixture was extracted with Et_2O and the combined organic layers were washed with brine and then were dried over Na_2SO_4. The solvents were removed *in vacuo* and the residue was then purified by flash column chromatography (pentane:diethyl ether, 12:1) to yield **14** as a colorless oil (3.24 g, 74%).

$[\alpha]_D^{20}$ = +22.2 (11 mM in $CHCl_3$); IR (neat): 3485, 2968, 1380, 1228, 1067 cm^{-1}; ^{1}H NMR (600 MHz, $CDCl_3$) δ: 5.95 (ddd, J = 17.3, 10.4, 7.1 Hz, 1H), 5.36 (m, 1H), 5.35 (apparent dt, J = 17.3, 1.0 Hz, 1H), 5.29 (apparent dt, J = 10.4, 1.0 Hz, 1H), 5.02 (brs, 1H), 4.70 (ddt, 7.1, 4.1, 1.0 Hz, 1H), 4.36–4.30 (m, 4H), 4.25 (d, J = 13.5 Hz, 1H) 3.54 (d, 2.9 Hz, 1H), 1.48 (s, 3H), 1.34 (s, 3H), 1.14–1.08 (m, 21H); ^{13}C NMR (151 MHz, $CDCl_3$) δ: 142.0, 138.1, 118.3, 109.9, 99.7, 79.1, 71.3, 70.8, 65.1, 64.1, 28.3, 21.8, 18.2(0), 18.1(8), 12.6; HRMS (ESI): m/z $[M + H]^+$ calcd for $C_{21}H_{40}ClO_4Si$, 419.2367; found, 419.2379.

Epoxide 15: To a solution of **14** (9.11 g, 21.7 mmol) in EtOH/H_2O (5/1, 150 mL) was added CsOH (50% w/w in H_2O, 21.0 mL, 109 mmol). The resulting mixture was heated to 80 °C and was stirred for 3 h, then cooled to room temperature. The mixture was extracted with Et_2O and the combined organic layers were washed with $NaHCO_3$ (aq.) and brine, then dried over Na_2SO_4. The solvents were removed *in vacuo* and the residue was then purified by flash column chromatography (pentane:diethyl ether, 10:1) to yield the TIPS protected epoxide as a colorless

oil (5.91 g, 71%). To a solution of this epoxide in THF (30 mL) was added tetrabutylammonium fluoride (19.1 mL, 1.0 M in THF, 19.1 mmol). The reaction was stirred at ambient temperature for 1 h and then was treated with H_2O. The mixture was extracted with Et_2O and the combined organic layers were washed with brine and then were dried over Na_2SO_4. The solvents were removed *in vacuo* and the residue was purified by flash column chromatography (pentane:ethyl acetate, 2:1) to yield **15** as a white solid (3.43 g, 98%).

mp: 60–61 °C; $[\alpha]_D^{20}$ = –15.0 (30 mM in $CHCl_3$); IR (neat): 3445, 2991, 1372, 1222, 1199, 1158, 1084, 1002 cm^{-1}; ^{1}H NMR (400 MHz, $CDCl_3$) δ: 6.00 (ddd, J = 17.4, 10.7, 4.5 Hz, 1H), 5.48 (dt, J = 17.4, 1.5 Hz, 1H), 5.28 (dt, J = 10.7, 1.5 Hz, 1H), 5.21–5.19 (m, 1H), 5.04–5.02 (m, 1H), 4.39 (d, J = 14.0 Hz, 1H), 4.29 (d, J = 14.0 Hz, 1H), 4.26 (d, J = 7.8 Hz, 1H), 4.18–4.16 (m, 1H), 3.26 (dd, J = 7.8, 4.2 Hz, 1H), 3.03 (dd, J = 7.8, 4.2 Hz, 1H), 2.24 (d, J = 3.8 Hz, 1H), 1.44 (s, 3H), 1.40 (s, 3H); ^{13}C NMR (101 MHz, $CDCl_3$) δ: 142.4, 135.9, 116.6, 109.1, 99.4, 70.9, 69.8, 64.0, 58.8, 57.3, 27.6, 21.4; HRMS (ESI): m/z $[M + Na]^+$ calcd for $C_{12}H_{18}O_4Na$, 249.1097; found, 249.1111.

Cyclohexenol 16: To a solution of **15** (300 mg, 1.33 mmol) in CH_2Cl_2 (40 mL) was added Stewart-Grubbs' catalyst (30 mg, 0.053 mmol). The mixture was heated at reflux under argon for 72 h. The reaction mixture was then cooled to room temperature and concentrated *in vacuo*. The residue was then purified by flash column chromatography (pentane:ethyl acetate, 1:1) to yield **16** as a white solid (240 mg, 91%).

mp: 81–82 °C; $[\alpha]_D^{20}$ = +95.0 (39 mM in $CHCl_3$); IR (neat): 3424, 2989, 1382, 1223, 1198, 1072, 1013 cm^{-1}; ^{1}H NMR (600 MHz, $CDCl_3$) δ: 5.47–5.46 (m, 1H), 4.82 (brs, 1H), 4.53 (brs, 1H), 4.37 (d, *J* = 14.4 Hz, 1H), 4.17 (d, *J* = 14.4 Hz, 1H), 3.44-3.43 (m, 1H), 3.39–3.37 (m, 1H), 2.28 (br. d, *J* = 4.4 Hz, 1H), 1.51 (s, 3H), 1.43 (s, 3H); ^{13}C NMR (151 MHz, $CDCl_3$) δ: 134.1, 118.0, 100.4, 65.4, 63.5, 62.5, 53.6, 51.9, 27.0, 21.3; HRMS (ESI): m/z $[M + H]^+$ calcd for $C_{10}H_{15}O_4$, 199.0965; found, 199.0973.

Carbonate 18: A mixture of Cs_2CO_3 (326 mg, 1.0 mmol) and powdered 3Å molecular sieves (160 mg) was heated under vaccum for 5 min, then blanketed with CO_2 (g) and cooled to room

temperature. A solution of **16** (198 mg, 1.0 mmol) in DMF (2 mL) was then added. The resulting light brown solution was heated to 45 °C, and maintained at this temperature for 18 h with stirring and then quenched with NH_4Cl (aq.). The resulting mixture was extracted with Et_2O and the combined organic layers were washed with brine and then dried over Na_2SO_4. The solvents were removed *in vacuo* and the residue was purified by flash column chromatography (pentane:ethyl acetate, 3:1) to yield **18** as a white solid (230 mg, 95%).

mp: 156–157 °C; $[\alpha]_D^{20}$ = +35.0 (7.8 mM in $CHCl_3$); IR (neat): 3479, 2942, 1803, 1383, 1163, 1043 cm^{-1}; ^1H NMR (400 MHz, $CDCl_3$) δ: 5.60-5.57 (m, 1H), 5.20-5.18 (m, 1H), 4.98 (dd, *J* = 6.8, 3.8 Hz, 1H), 4.66–4.65 (m, 1H), 4.52–4.79 (m, 1H), 4.43 (t, *J* = 3.8 Hz, 1H), 4.21 (dd, *J* = 14.8, 0.8 Hz, 1H), 2.69 (brs, 1H), 1.55 (s, 3H), 1.44 (s, 3H); ^{13}C NMR (101 MHz, $CDCl_3$) δ: 153.9, 136.8, 114.7, 100.2, 74.4, 72.1, 65.9, 64.7, 62.5, 27.6, 21.0; HRMS (ESI): m/z $[M + H]^+$ calcd for $C_{11}H_{15}O_6$, 243.0863; found, 243.0863.

Carbasugar 3b: To a solution of **18** (48.5 mg, 0.2 mmol) in THF/methanol (1/1, 2 mL) at 0 °C was added K_2CO_3 (27.6 mg, 0.2 mmol). The resulting mixture was stirred at 0 °C for 1 h and then filtered through a pad of silica gel. The solvents were removed *in vacuo* and the residue was dissolved in DMF (0.8 mL). Quinuclidine (111 mg, 1.0 mmol) and 4Å molecular sieves (10 beads) were added and the resulting solution was stirred at ambient temperature for 30 min. Then a solution of 2,4-dinitrofluorobenzene (37.2 mg, 0.2 mmol) in DMF (0.2 mL) was added dropwise. The reaction mixture was then stirred at ambient temperature for 12 h and then cooled to 0 °C. Methanol (2 mL) was added, followed by aqueous HCl (1.0 M) to pH~3. The resultant mixture was stirred at 0 °C for 20 min and was then quickly neutralized by adding trimethylamine, and subsequently purified by flash column chromatography (CH_2Cl_2: methanol, 12:1) to yield **3b** as a white foam (12.3 mg, 18%).

$[\alpha]_D^{20}$ = +121.7 (9.1 mM in CH_3OH); IR (neat): 3361, 2930, 1611, 1520, 1348, 1076 cm^{-1}; ^1H NMR (600 MHz, CD_3OD) δ: 8.70 (d, J = 2.8 Hz, 1H), 8.45 (dd, J = 9.4, 2.8 Hz, 1H), 7.69 (d, J = 9.4 Hz, 1H), 5.99–5.98 (m, 1H), 5.42 (t, J = 4.2 Hz, 1H), 4.29 (d, J = 4.2 Hz, 1H), 4.22 (d, J = 15.0 Hz, 1H), 4.19 (dd, J = 9.7, 3.8 Hz, 1H), 4.16 (d, J = 15.0 Hz, 1H), 4.05 (dd, J = 9.7, 4.2 Hz,

1H); ^{13}C NMR (151 MHz, CD_3OD) δ: 158.1, 147.0, 141.3, 140.9, 129.7, 122.5, 118.8, 117.9, 77.8, 70.4, 69.6, 68.1, 63.5; HRMS (ESI): m/z $[M + Na]^+$ calcd for $C_{13}H_{14}N_2NaO_9$, 365.0592; found, 365.0583.

Alkene 20: To a solution of **9** (260 mg, 1.0 mmol) in DMF (10 mL) at 5 °C were added Selectfluor® (350 mg, 1.0 mmol) and (*R*)-proline (115 mg, 1.0 mmol). The mixture was stirred at 5 °C for 1 h, treated with H_2O, then extracted with Et_2O. The combined organic layers were washed with brine and then dried over Na_2SO_4. The solvents were removed *in vacuo* and the residue was redissolved in CH_2Cl_2 (5 mL). (*R*)-proline (92 mg, 0.8 mmol) and 2,2-dimethyl-1,3-dioxan-5-one (**12**; 156 mg, 1.2 mmol) were then added at 0 °C. The mixture was warmed to room temperature and stirred for 48 h. The resulting mixture was then was treated with H_2O and extracted with Et_2O. The combined organic layers were washed with brine and then dried over Na_2SO_4. The solvents were removed *in vacuo* and the residue was dissolved in THF (3 mL). In another flask LiHMDS (2.0 mL, 1.0 M in THF, 2.0 mmol) was added dropwise to a cooled (–78 °C) solution of 5-(methanesulfonyl)-1-phenyl-1*H*-tetrazole (444 mg, 2.0 mmol) in THF (7 mL) and stirred at –78 °C for 30 min. Then the above solution of ketone **19** in THF (3 mL) was added dropwise at –78 °C and the mixture was stirred for another 1 h before quenching with H_2O. The mixture was extracted with Et_2O and the combined organic layers were washed with brine and then dried over Na_2SO_4. The solvents were removed *in vacuo* and the residue was purified by flash column chromatography (pentane:diethyl ether, 15:1) to yield **20** as a colorless oil (161 mg, 40%).

$[\alpha]_D^{20}$ = +19.8 (11 mM in $CHCl_3$); IR (neat): 3478, 2944, 2868, 1464, 1381, 1096, 1071 cm^{-1}; ^{1}H NMR (500 MHz, $CDCl_3$): δ 5.88 (ddd, J = 17.2, 10.5, 6.3 Hz, 1H), 5.42 (apparent d, J = 17.2 Hz, 1H), 5.33–5.31 (m, 2H), 5.04 (brs, 1H), 4.86–4.83 (m, 1H), 4.67 (dd, J = 44.2, 3.7 Hz, 1H), 4.43 (d, J = 8.5 Hz, 1H), 4.36 (d, J = 13.0 Hz, 1H), 4.26 (d, J = 13.2 Hz, 1H), 4.14 (ddd, J = 29.0, 8.7, 2.0 Hz, 1H), 4.11-4.09 (m, 1H), 1.49 (s, 3H), 1.34 (s, 3H), 1.11–1.05 (m, 21H); ^{13}C NMR (101 MHz, $CDCl_3$) δ 142.1, 136.4 (d, $J_{C\text{-}F}$ = 7.8 Hz), 118.2 (d, $J_{C\text{-}F}$ = 1.5 Hz), 109.9, 99.6, 90.1 (d, $J_{C\text{-}F}$ = 185.1 Hz), 76.6 (d, $J_{C\text{-}F}$ = 22.8 Hz), 70.8 (d, $J_{C\text{-}F}$ = 18.3 Hz), 70.5(d, $J_{C\text{-}F}$ = 3.8 Hz), 65.1, 28.3,

22.0, 18.03, 18.02, 12.4; HRMS (ESI): m/z $[M + Na]^+$ calcd for $C_{21}H_{39}FNaO_4Si$, 425.2494; found, 425.2495.

Acetate 21: To a solution of **20** (201 mg, 0.5 mmol) in CH_2Cl_2 (5 mL) at ambient temperature was added triethylamine (139 μL, 1.0 mmol), acetic anhydride (71 μL, 0.75 mmol), and 4-dimethylaminopyridine (6.1 mg, 0.05 mmol). The reaction mixture was stirred at ambient temperature for 48 h and then treated with NH_4Cl (aq.). The resulting mixture was extracted with Et_2O and the combined organic layers were washed with brine and then dried over Na_2SO_4. The solvents were removed *in vacuo* and the residue was purified by flash column chromatography (pentane:diethyl ether, 8:1) to yield **21** as a colorless oil (186 mg, 84%).

$[\alpha]_D^{20}$ = +43.2 (20 mM in $CHCl_3$); IR (neat): 2944, 2868, 1749, 1464, 1371, 1232, 1094, 1040 cm^{-1}; ^1H NMR (500 MHz, $CDCl_3$): δ 5.88 (ddd, *J* = 17.2, 10.1, 7.9 Hz, 1H), 5.44 (ddd, *J* = 27.1, 9.0, 1.0 Hz, 1H), 5.28 (d, *J* = 17.2 Hz, 1H), 5.26 (d, *J* = 10.1 Hz, 1H), 4.99 (brs, 1H), 4.97 (brs, 1H), 4.81 (ddd, *J* = 44.9, 6.0, 1.0 Hz, 1H), 4.55-4.52 (m, 1H), 4.45 (d, *J* = 8.9 Hz, 1H), 4.37-4.32 (m, 1H), 4.27 (d, *J* = 13.8 Hz, 1H), 2.02 (s, 3H), 1.45 (s, 3H), 1.38 (s, 3H), 1.05 (brs, 21H); ^{13}C NMR (101 MHz, $CDCl_3$) δ 169.8, 141.1, 137.2 (d, $J_{C\text{-}F}$ = 4.0 Hz), 118.5 (d, $J_{C\text{-}F}$ = 1.6 Hz), 111.5, 99.6, 91.5 (d, $J_{C\text{-}F}$ = 184.5 Hz), 74.0 (d, $J_{C\text{-}F}$ = 25.4 Hz), 72.1(d, $J_{C\text{-}F}$ = 4.0 Hz), 70.4 (d, $J_{C\text{-}F}$ = 16.3 Hz), 64.0, 28.8, 24.9, 21.1, 18.2, 12.7; HRMS (ESI): m/z $[M + Na]^+$ calcd for $C_{23}H_{41}FNaO_5Si$, 467.2600; found, 467.2595.

Cyclohexenol 22: To a solution of **21** (160 mg, 0.36 mmol) in THF (3.6 mL) at 0 °C was added a solution of tetrabutylammonium fluoride (0.72 mL, 1.0 M in THF, 0.72 mmol) and acetic acid (43 μL, 0.72 mmol). The reaction mixture was stirred at ambient temperature for 48 h and then was treated with H_2O. The mixture was extracted with Et_2O and the combined organic layers were washed with brine and then were dried over Na_2SO_4. The solvents were removed *in vacuo* and the residue was purified by flash column chromatography (pentane:ethyl acetate, 3:1) to yield a 10:1 mixture of the desired deprotection product and acyl migration compound. The mixture was redissolved in CH_2Cl_2 (18 mL) and Grubbs' II catalyst (31 mg, 0.036 mmol) was added. The mixture was heated to 40 °C under argon and maintained at that temperature for 1 h. The reaction

was cooled to room temperature and concentrated *in vacuo*. The residue was then purified by flash column chromatography (pentane:ethyl acetate, 1:1.5) to yield **22** as a yellow oil (82 mg, 88%).

$[\alpha]_D^{20}$ = +104.3 (30 mM in $CHCl_3$); IR (neat): 3449, 2992, 2926, 1749, 1377, 1234, 1097, 1064 cm^{-1}; ^{1}H NMR (400 MHz, $CDCl_3$): δ 5.59 (ddd, J = 6.0, 4.2, 1.8 Hz, 1H), 5.53 (brs, 1H), 4.89 (dddd, J = 48.9, 5.8, 3.4, 1.1 Hz, 1H), 4.71 (brs, 1H), 4.51–4.47 (m, 1H), 4.45-4.37 (m, 1H), 4.15 (d, J = 14.4 Hz, 1H), 2.09 (brs, 1H), 2.09 (s, 3H), 1.51 (s, 3H), 1.37 (s, 3H); ^{13}C NMR (101 MHz, $CDCl_3$) δ 170.1, 133.1, 120.3 (d, $J_{C\text{-}F}$ = 2.1 Hz), 99.7, 88.1 (d, $J_{C\text{-}F}$ = 174.9 Hz), 68.0 (d, $J_{C\text{-}F}$ = 26.3 Hz), 65.3 (d, $J_{C\text{-}F}$ = 18.5 Hz), 64.4 (d, $J_{C\text{-}F}$ = 3.5 Hz), 63.3, 28.3, 20.9, 20.1; HRMS (ESI): m/z $[M + Na]^+$ calcd for $C_{12}H_{17}FNaO_5$, 283.0952; found, 283.0954.

Carbasugar 4: To a solution of **22** (9.4 mg, 0.036 mmol) in DMF (0.18 mL) was added quinuclidine (20 mg, 0.18 mmol) and 4Å molecular sieves (2 beads). The resulting solution was stirred at ambient temperature for 30 min. Then a solution of 2,4-dinitrofluorobenzene (7.4 mg, 0.040 mmol) in DMF (0.1 mL) was added dropwise. The reaction mixture was stirred at ambient temperature for 12 h and then methanol (0.4 mL) was added, followed by K_2CO_3 (7.5 mg, 0.054 mmol). The resultant mixture was stirred at ambient temperature for another 1 h and then cooled to 0 °C, acidified with aqueous HCl (1.0 M) to pH~3. The reaction mixture was stirred at 0 °C for 15 min and was then quickly neutralized by adding trimethylamine, and purified by flash column chromatography (CH_2Cl_2: methanol, 20:1) to yield **4** as a white solid (8.2 mg, 66%).

mp: 158–159 °C; $[\alpha]_D^{20}$ +190.0 (4.9 mM in CH_3OH); IR (neat): 3363, 2926, 1605, 1532, 1347, 1279, 1068 cm^{-1}; ^{1}H NMR (600 MHz, CD_3OD): δ 8.69 (d, *J* = 1.5 Hz, 1H), 8.46 (dd, *J* = 9.4, 1.5 Hz, 1H), 7.69 (d, *J* = 9.6 Hz, 1H), 5.98 (brs, 1H), 5.64 (dd, *J* = 4.1, 3.6 Hz, 1H), 5.01 (ddd, *J* = 49.0, 10.2, 3.6 Hz, 1H), 4.31 (apparent t, *J* = 4.0 Hz, 1H), 4.28-4.24 (m, 1H), 4.24 (d, *J* = 15.3 Hz, 1H), 4.15 (d, *J* = 15.3 Hz, 1H); ^{13}C NMR (151 MHz, CD_3OD) δ 157.4, 148.2, 141.7, 141.0, 129.7, 122.3, 117.7, 117.5 (d, $J_{C\text{-}F}$ = 4.4 Hz), 90.4 (d, $J_{C\text{-}F}$ = 185.3 Hz), 76.3 (d, $J_{C\text{-}F}$ = 16.5 Hz), 68.5 (d, $J_{C\text{-}F}$ = 10.3 Hz), 68.4, 63.4; HRMS (ESI): m/z $[M + Na]^+$ calcd for $C_{13}H_{13}FN_2NaO_2$: 367.0548; found: 367.0550.

2,4-Dinitrophenyl 2-deoxy-2-fluoro-α-D-galactopyranoside (7): The peracetylated material was separated from the major β-anomer, which was made by the methods reported by Withers and co-workers,[4] by preparative TLC. Deacetylation was performed using the same conditions as reported for the β-anomer.[4] IR (neat): 3360, 2938, 1611, 1542, 1323 cm^{-1}; ^{1}H NMR (600 MHz, CD_3OD): δ 8.77 (d, J = 2.7 Hz, 1H), 8.49 (dd, J = 9.4, 2.7 Hz, 1H), 7.74 (d, J = 9.4 Hz, 1H), 6.22 (d, J = 3.5 Hz, 1H), 4.86 (ddd, J = 48.9, 10.2, 3.5 Hz, 1H), 4.21 (apparent dt, J = 10.1, 3.3 Hz, 1H), 4.06 (t, J = 3.4 Hz, 1H), 3.91 (t, J = 5.8 Hz, 1H), 3.73-3.67 (m, 2H); ^{13}C NMR (151 MHz, CD_3OD) δ 155.0, 142.7, 141.1, 129.9, 122.4, 118.7, 97.6 (d, J_{C-F} = 22.2 Hz), 89.3 (d, J_{C-F} = 188.4 Hz), 75.2, 71.2 (d, J_{C-F} = 9.5 Hz), 69.4 (d, J_{C-F} = 17.4 Hz), 62.2; HRMS (ESI): m/z $[M + Na]^+$ calcd for $C_{12}H_{13}FN_2NaO_9$: 371.0497; found: 371.0490.

Kinetics of Reactivation for *Tm*GalA Inactivated by Cyclophellitol 8.

A solution of (total volume of 50 μL) WT-TmGalA (4.6 μM) in 50 mM HEPES buffer (pH 7.4) that contained 1 mg/mL BSA and cyclophellitol **8** (1 mM) was incubated for 16 hours at 37 °C. After incubation, excess inactivator was removed by filtration using a 10K molecular weight cutoff centrifugal filter and washing three times with 300 μL buffer (50 mM HEPES, pH 7.4) at 4 °C to give a final sample volume of 50 μL. The filtered sample was added to reactivation buffer (final volume 200 μL 50 mM HEPES, pH 7.4, with 1 mg/mL BSA) and this solution was incubated at 37 °C. α-Galactosidase activity was measured periodically by adding an aliquot (10 μL) of the reaction mixture to a pre-equilibrated solution of pNP α-galactoside (250 μM, 490 μL) in HEPES buffer (50 mM, pH 7.4) containing 1 mg/mL BSA and monitoring the change in absorbance at 400 nm using a Cary Eclipse UV spectrophotometer. No increase in enzyme activity was observed over a 30 h time period.

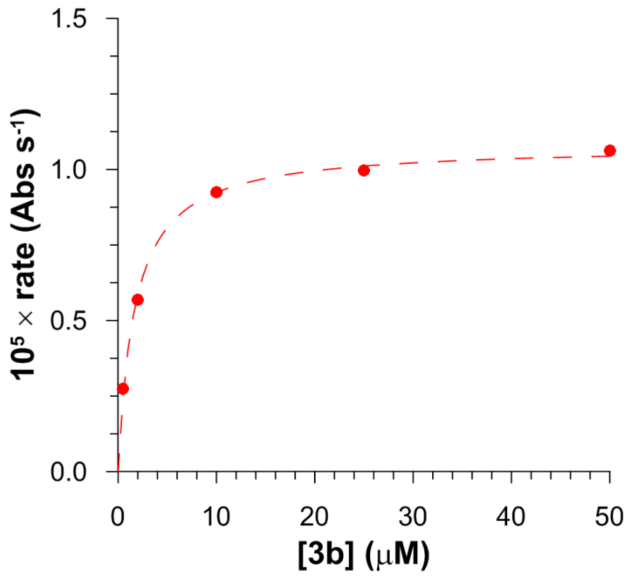

Supplementary Figure 1. The Michaelis–Menten plot for the *Tm*GalA-catalyzed hydrolysis of **3b**. Conditions for all experiments were T = 37 °C in 50 mM HEPES buffer, pH 7.4. The dashed line is the nonlinear least squares fits to a Michaelis–Menten equation.

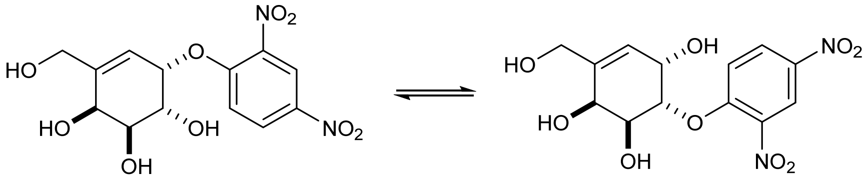

Supplementary Figure 2. Equilibration observed by ^{1}H NMR spectroscopy for the intramolecular migration of the 2,4-dinitrophenyl group in **3b**.

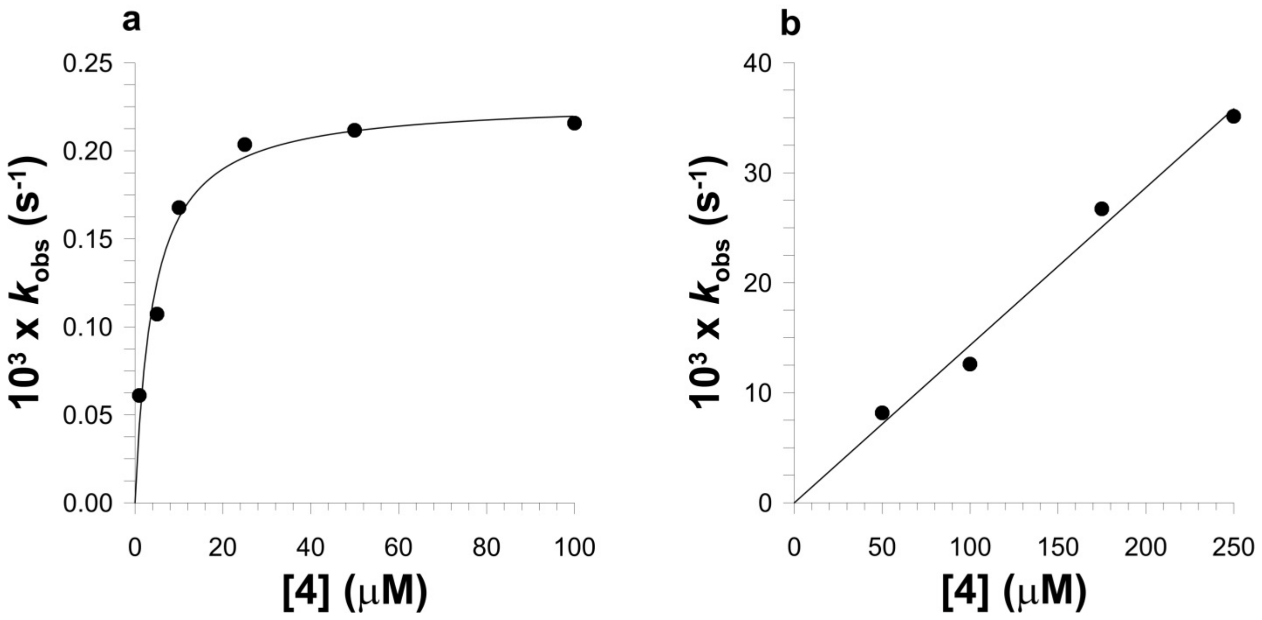

Supplementary Figure 3. Kinetics for the reactions catalyzed bt *Tm*GalA on compound **4**. a) Michaelis-Menten plot for the *Tm*GalA-catalyzed hydrolysis of **4**. The solid line is the nonlinear least squares fits to a standard Micahelis-Menten equation. b) A plot of the first-order rate constants for loss of *Tm*GalA activity as a function of concentration of covalent inhibitor **4**. Conditions for all experiments were T = 37 °C in 50 mM HEPES buffer, pH 7.4. The dashed line is the linear least squares fits to a straight line.

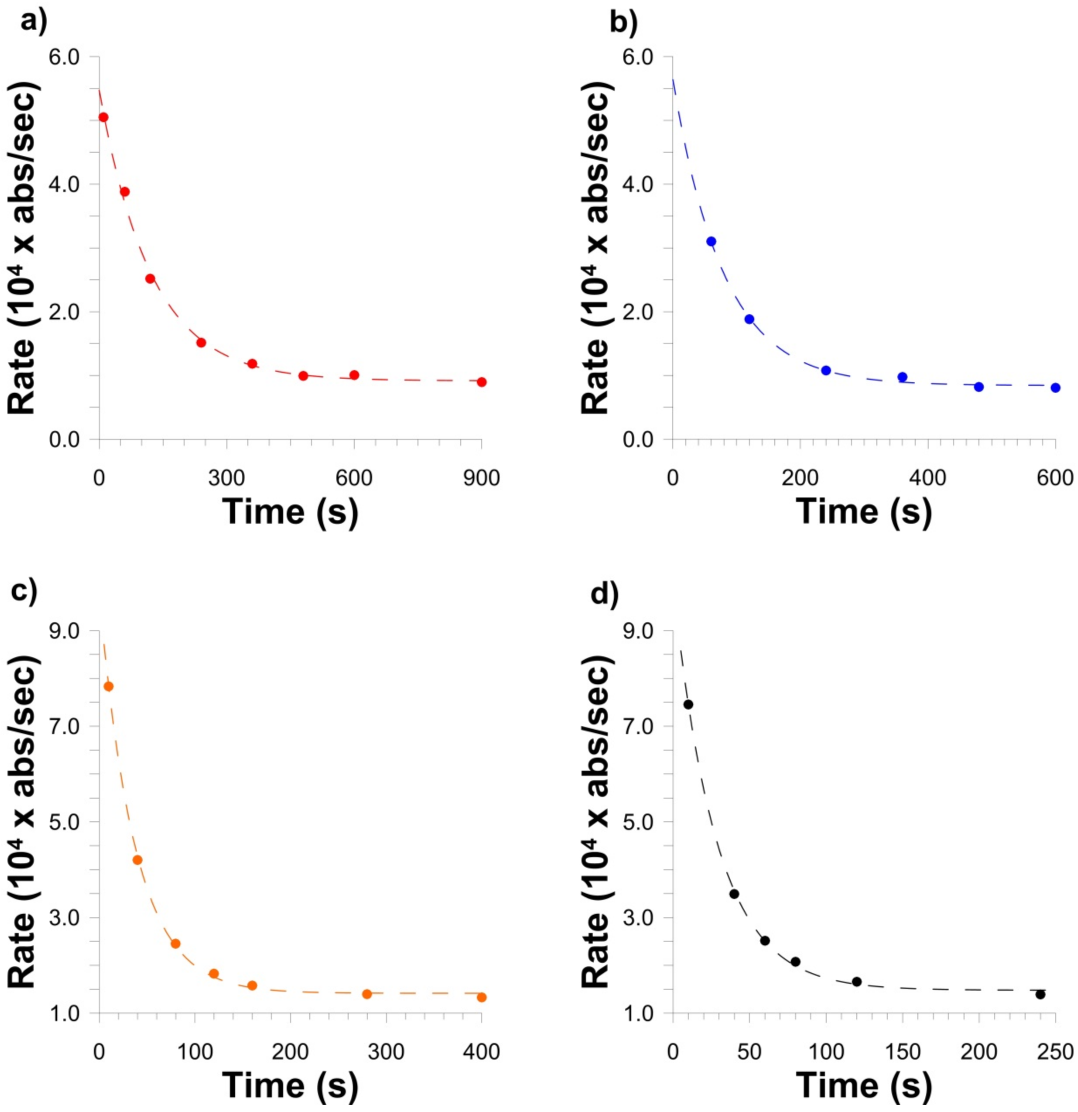

Supplementary Figure 4: Reaction kinetics for the inactivation of *Tm*GalA by compound **4**. Plots show enzyme activity versus incubation time. a) [**4**] = 50 μM; b) [**4**] = 100 μM; c) [**4**] = 175 μM; and d) [**4**] = 250 μM. Conditions for all experiments are *T* = 37 °C in 50 mM HEPES buffer, pH 7.4. The dashed lines are the nonlinear least squares fits to a standard first-order rate equation.

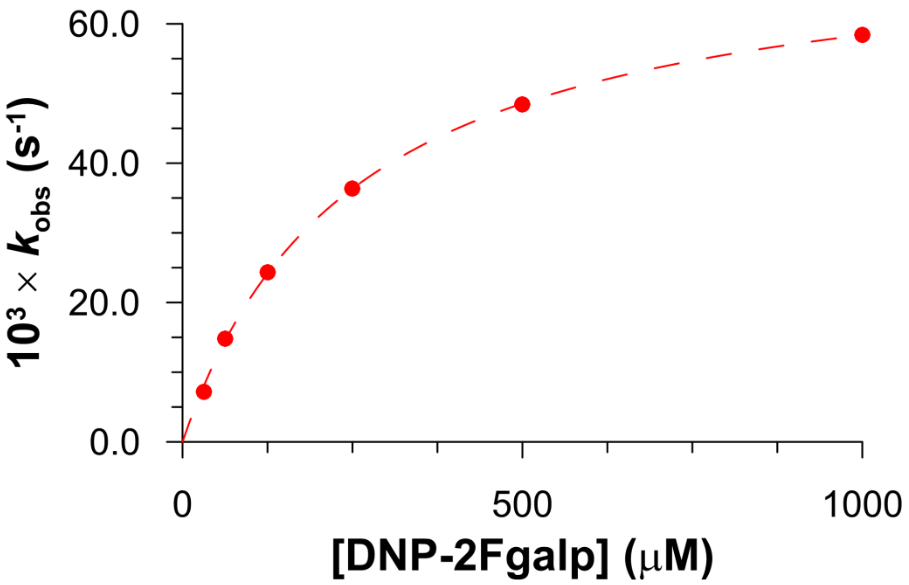

Supplementary Figure 5. Kinetics for the hydrolysis of 2,4-dinitrophenyl 2-deoxy-2-fluoro-α-D-galactopyranoside by *Tm*GalA. Conditions for all experiments were T = 37 °C in 50 mM HEPES buffer, pH 7.4. The dashed line is the nonlinear least squares fit to a standard Michaelis-Menten equation.

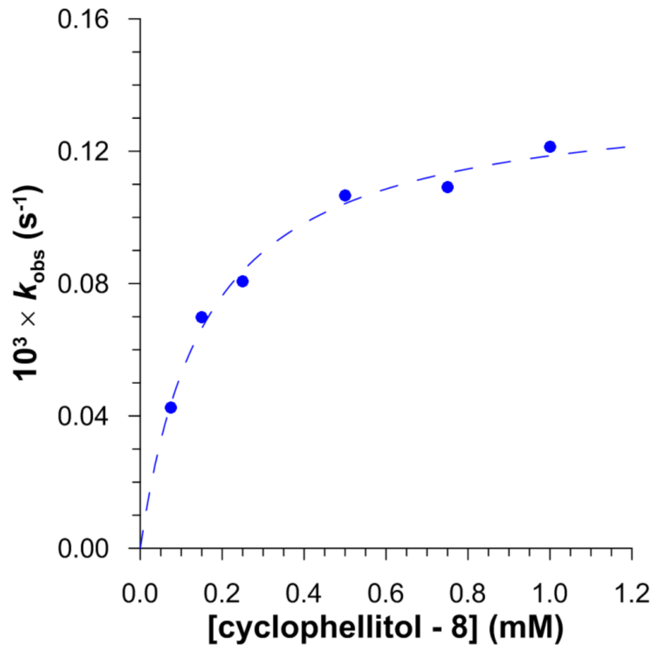

Supplementary Figure 6. Michaelis Menten plot for the inactivation of *Tm*GalA by cyclophellitol **8**. Conditions for all experiments were T = 37 °C in 50 mM HEPES buffer, pH 7.4. The dashed line is the nonlinear least squares fit to a standard Michaelis-Menten equation.

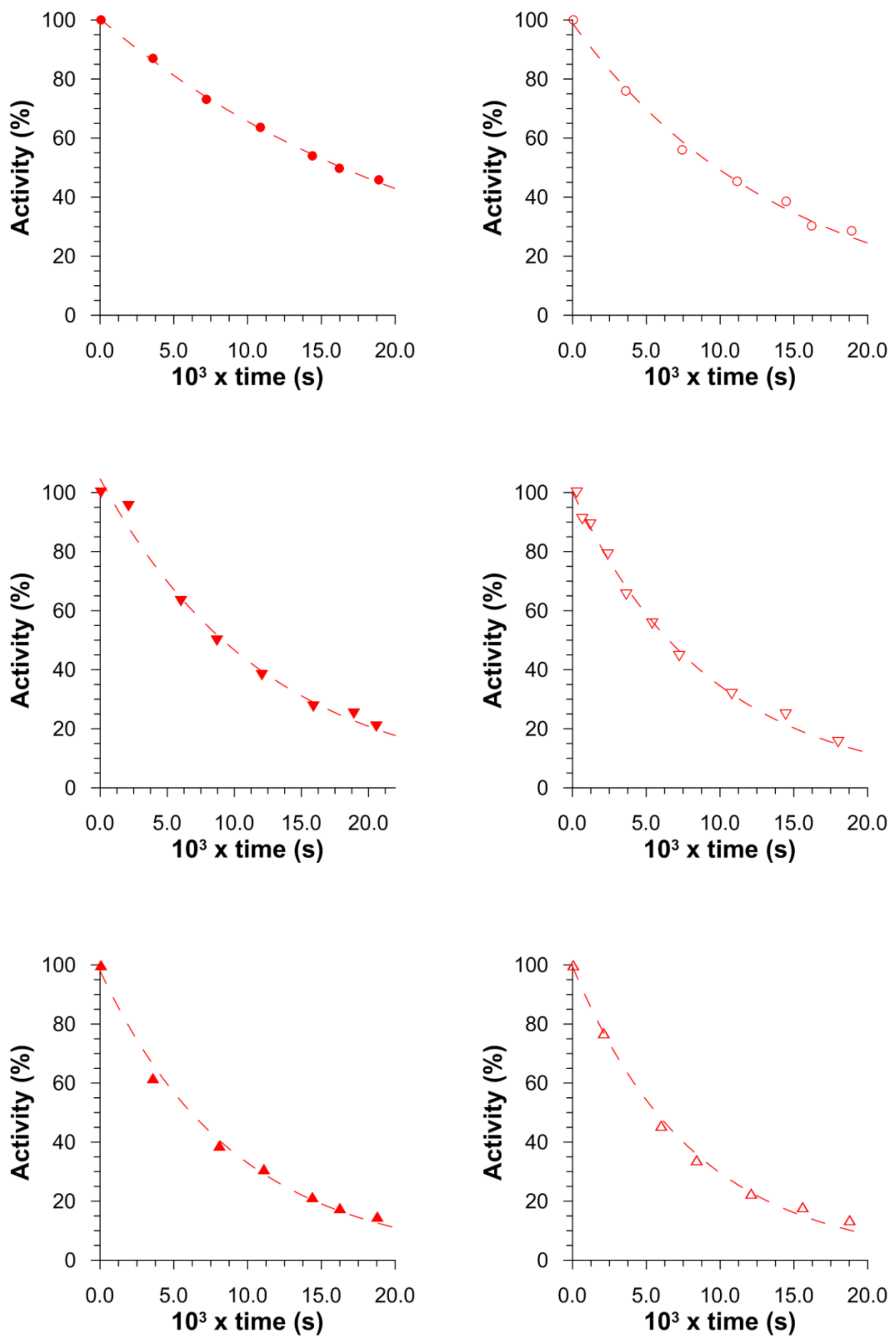

Supplementary Figure 7. Time dependent loss of *Tm*GalA activity during incubation with various concentrations of cyclophellitol **8**, solid circles (75 μM); hollow circles (150 μM); solid downward triangles (250 μM); hollow downward triangles (500 μM); solid upward triangles (750 μM); and hollow upward triangles (1000 μM). Conditions were T = 37 °C in 50 mM HEPES buffer, pH 7.4. Dashed lines are the nonlinear least squares fit to a standard first order equation.

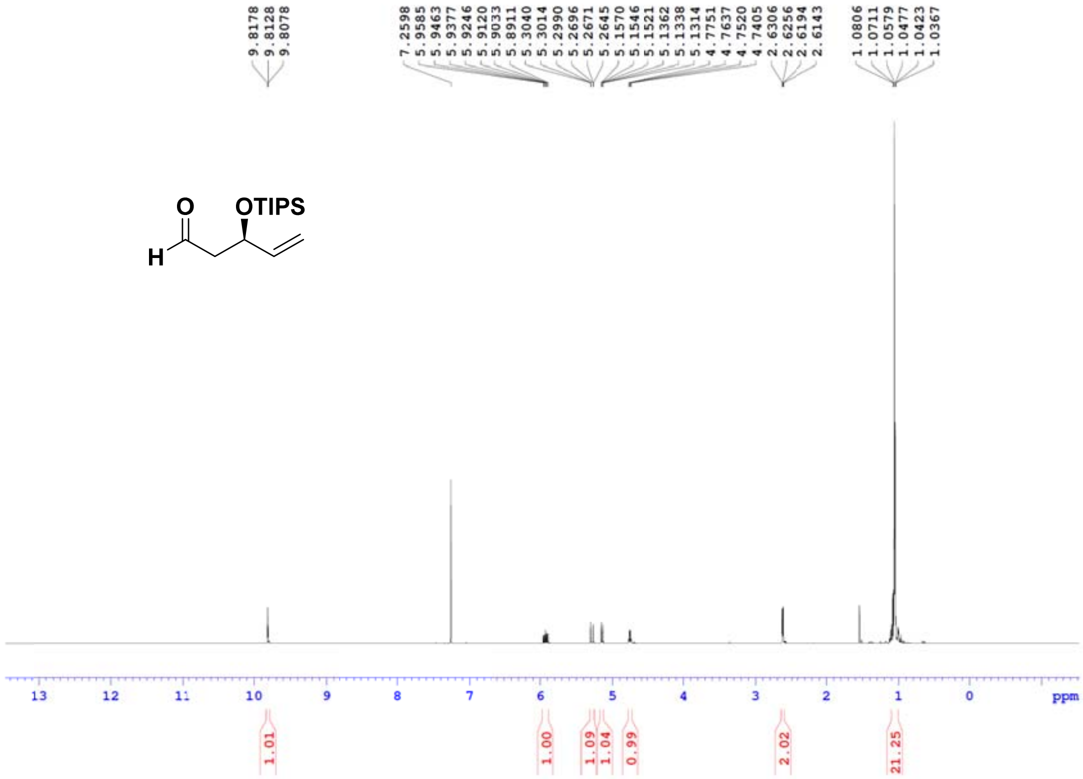

Supplementary Figure 8: ^{1}H NMR spectrum for **9** in $CDCl_3$.

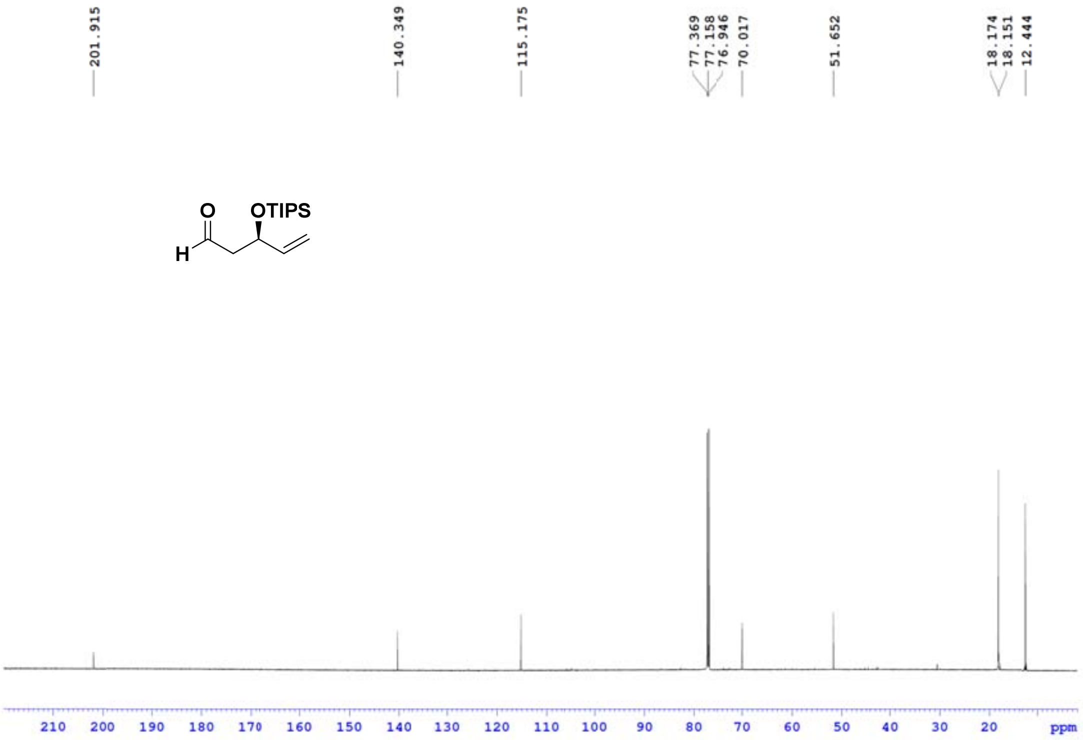

Supplementary Figure 9: ^{13}C NMR spectrum for **9** in $CDCl_3$.

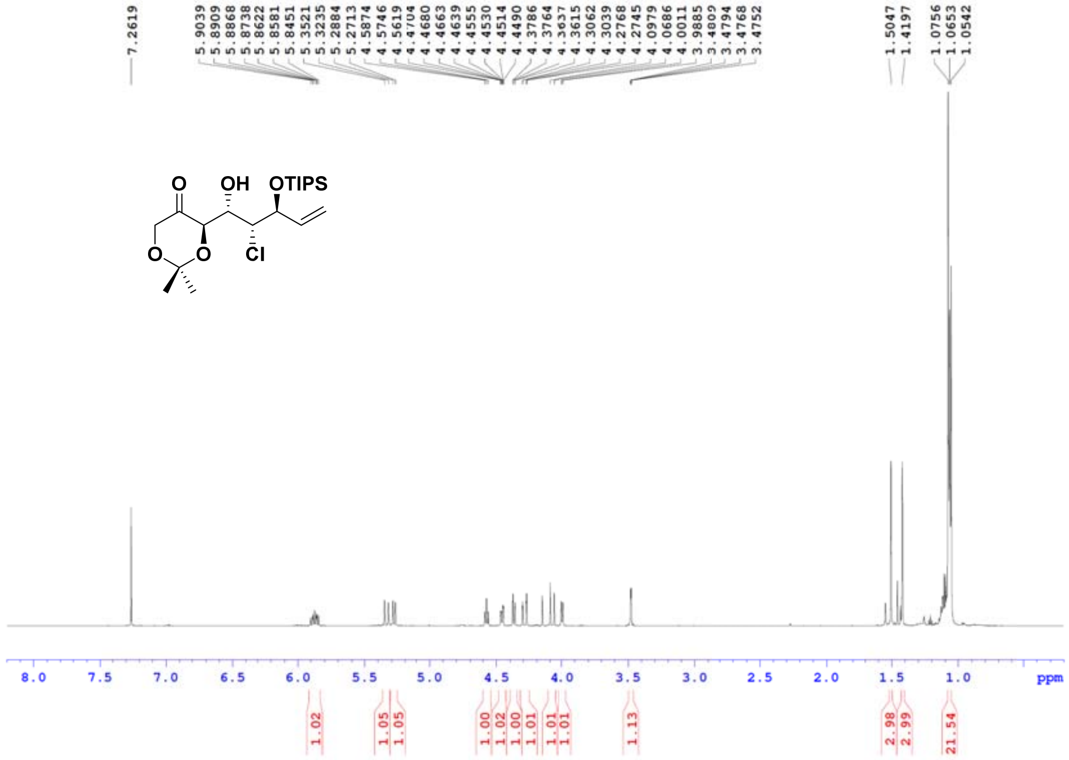

Supplementary Figure 10: ^{1}H NMR spectrum for **13** in $CDCl_3$.

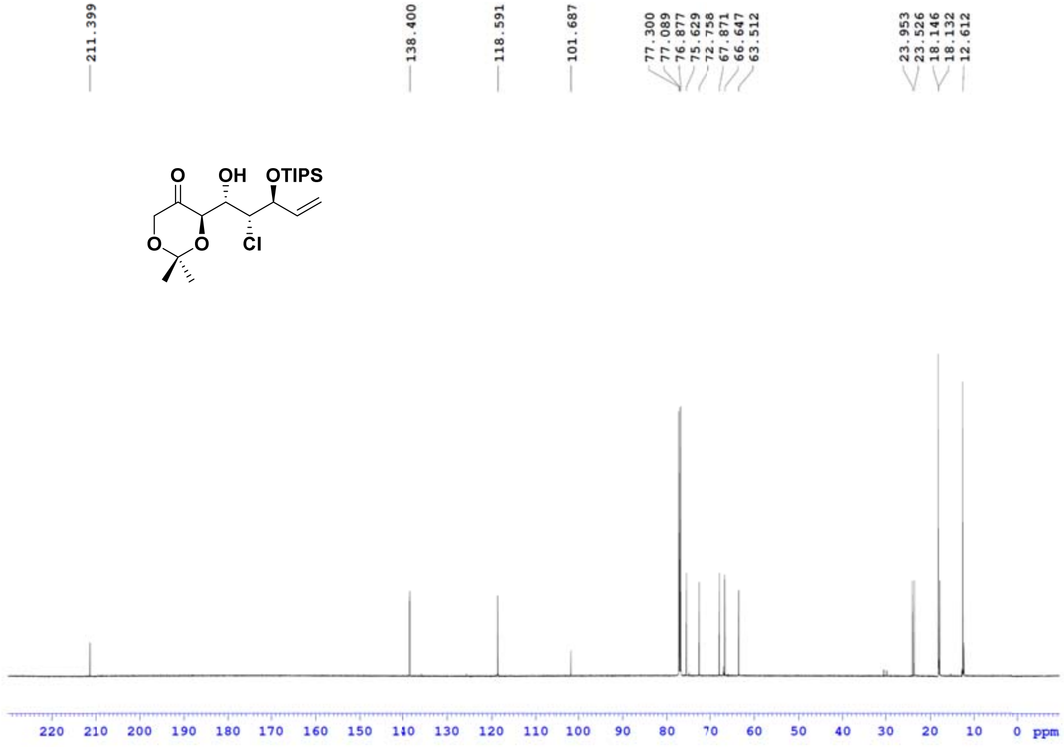

Supplementary Figure 11: ^{13}C NMR spectrum for **13** in $CDCl_3$.

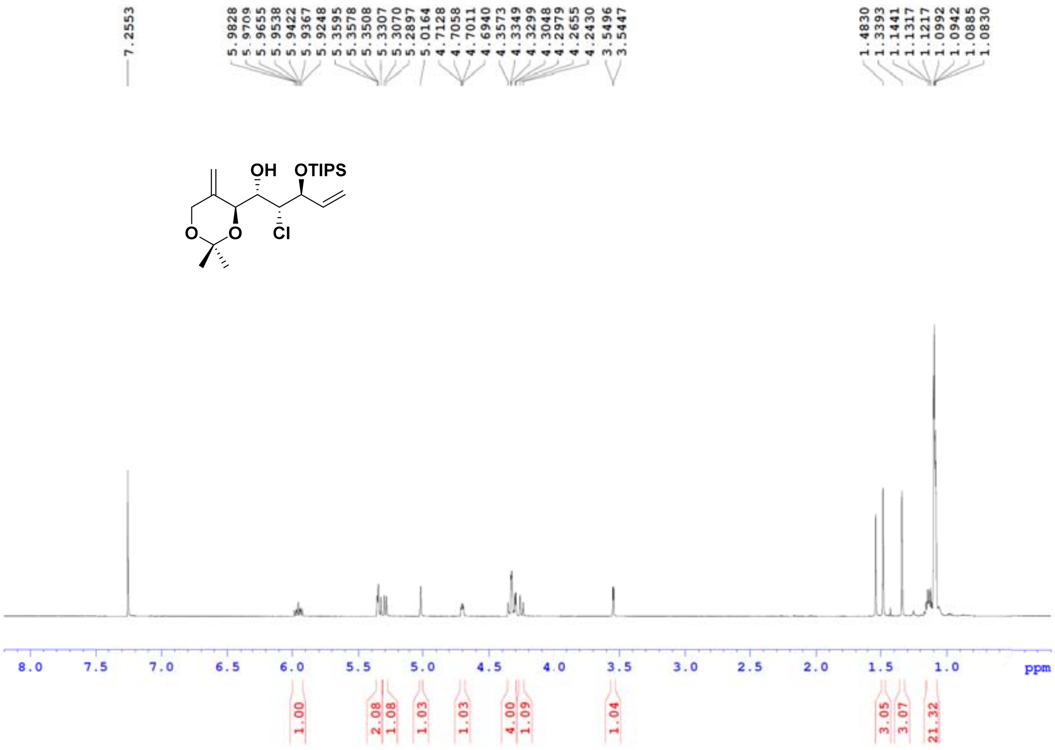

Supplementary Figure 12: ^{1}H NMR spectrum for **14** in $CDCl_3$.

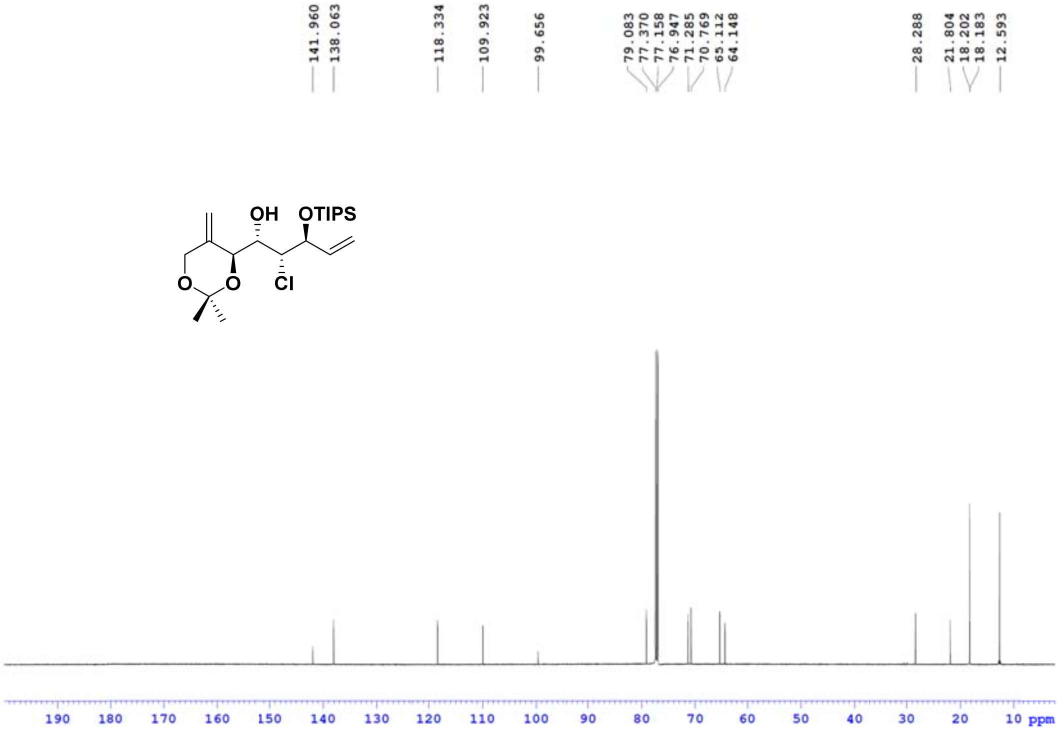

Supplementary Figure 13: ^{13}C NMR spectrum for **14** in $CDCl_3$.

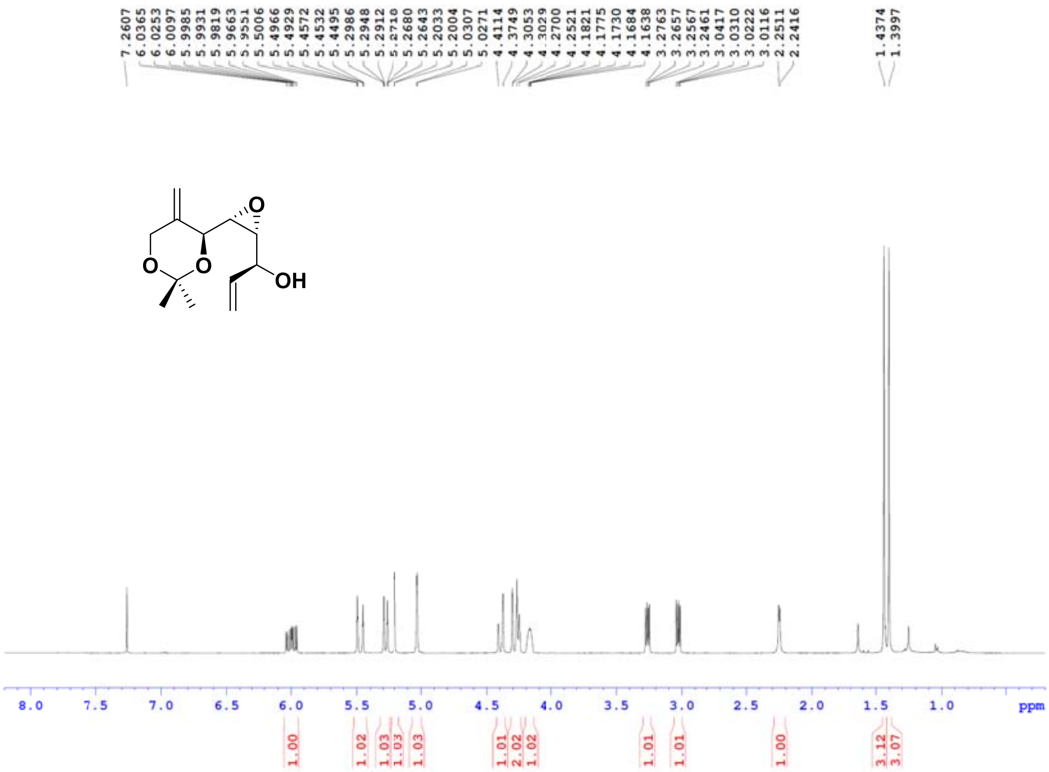

Supplementary Figure 14: ^{1}H NMR spectrum for **15** in $CDCl_3$.

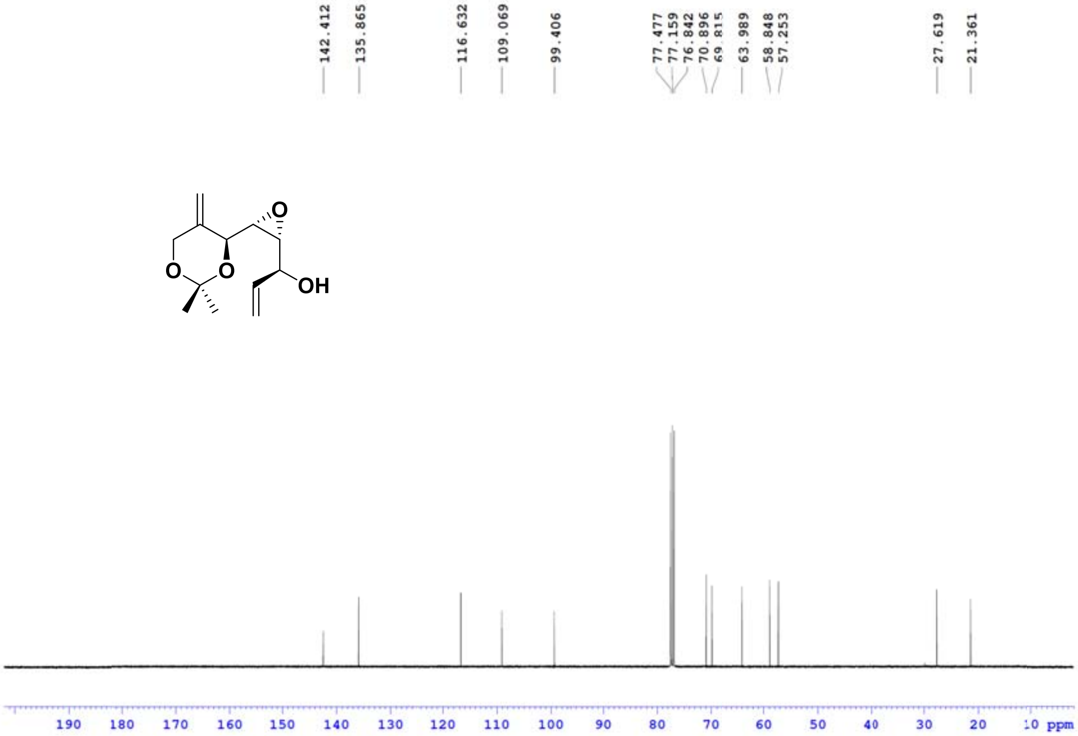

Supplementary Figure 15: ^{13}C NMR spectrum for **15** in $CDCl_3$.

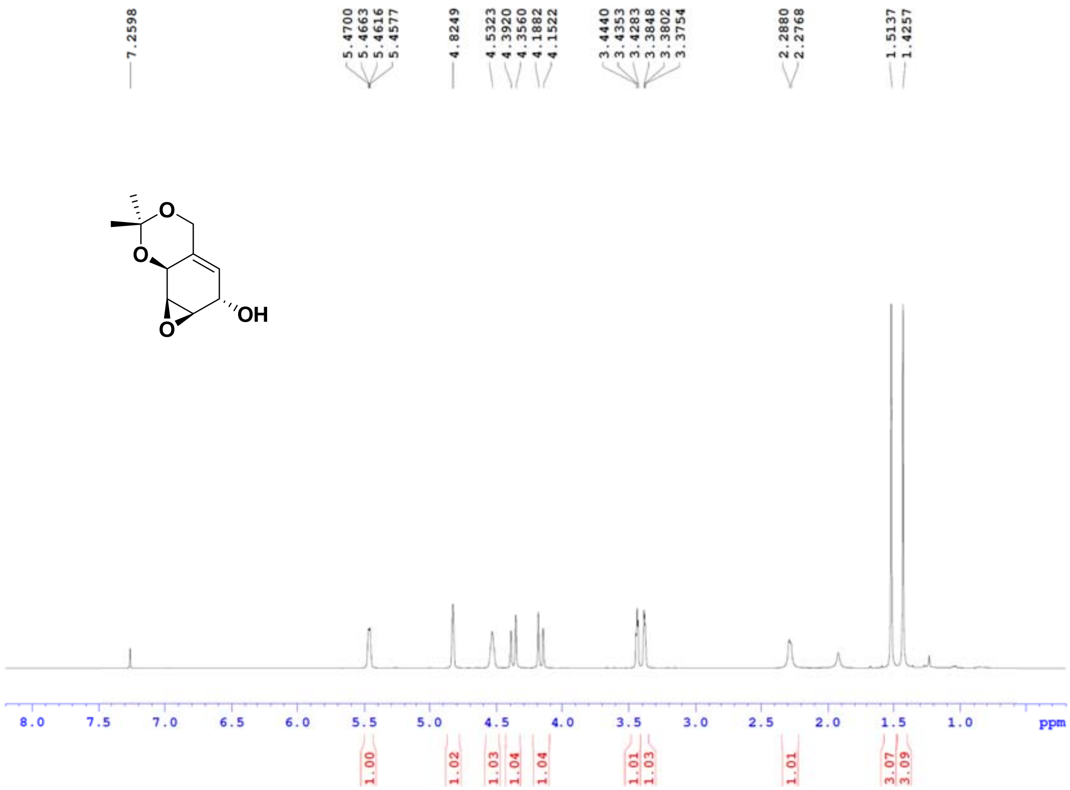

Supplementary Figure 16: ^{1}H NMR spectrum for **16** in $CDCl_3$.

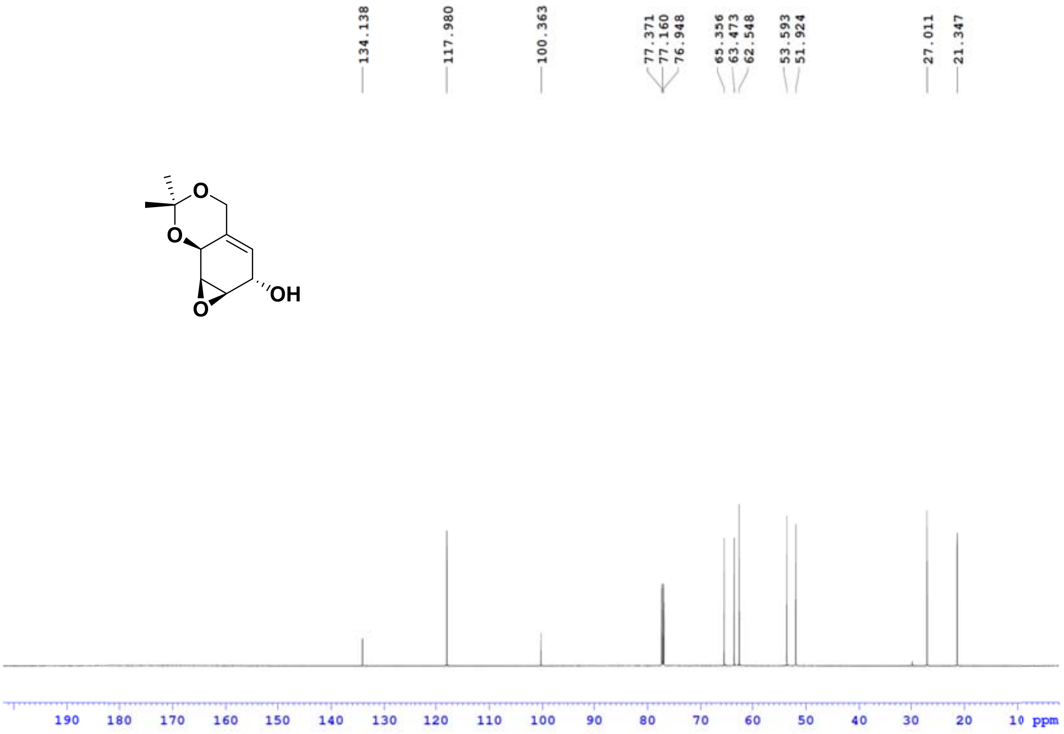

Supplementary Figure 17: ^{13}C NMR spectrum for **16** in $CDCl_3$.

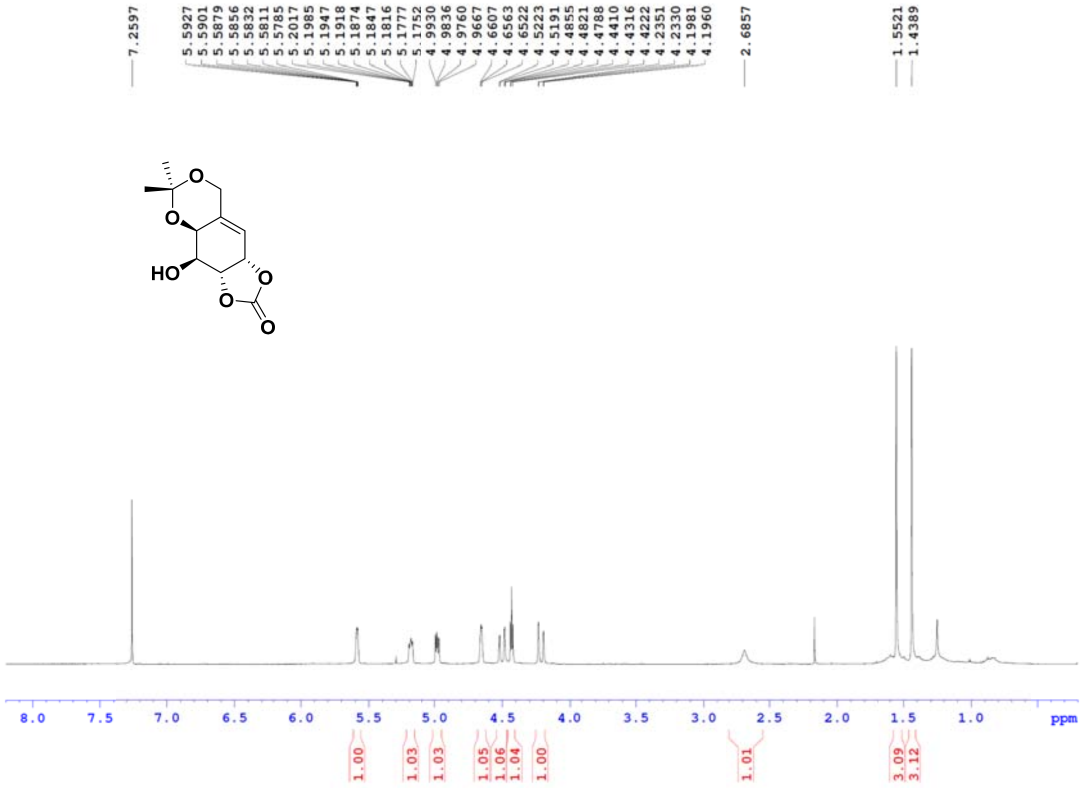

Supplementary Figure 18: ^{1}H NMR spectrum for **18** in $CDCl_3$.

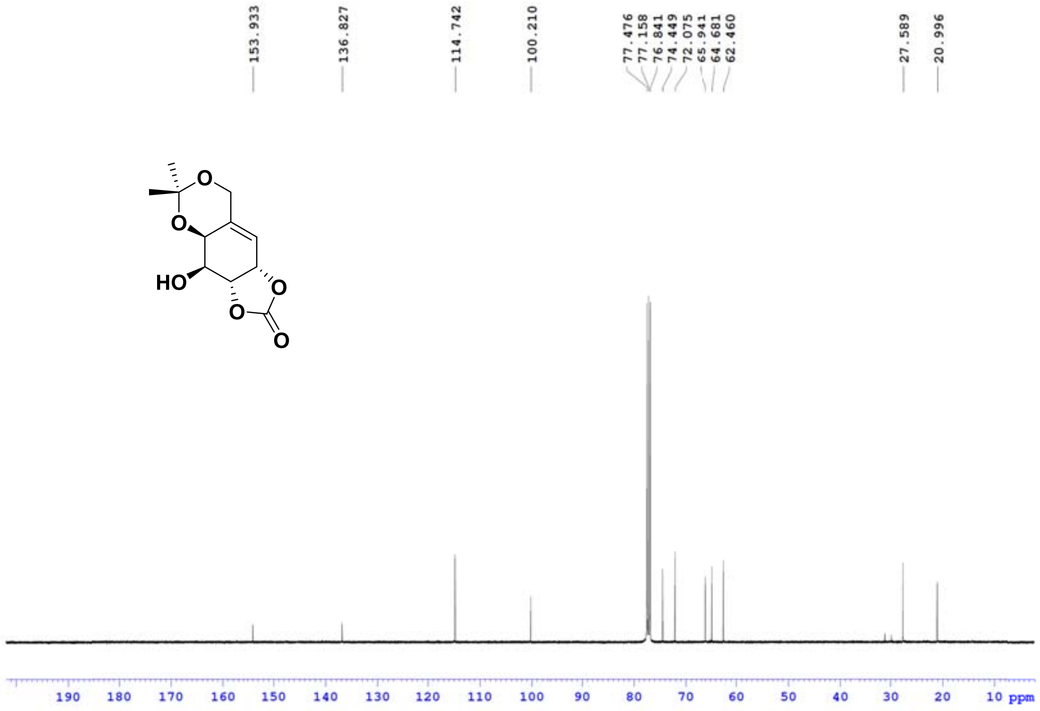

Supplementary Figure 19: ^{13}C NMR spectrum for **18** in $CDCl_3$.

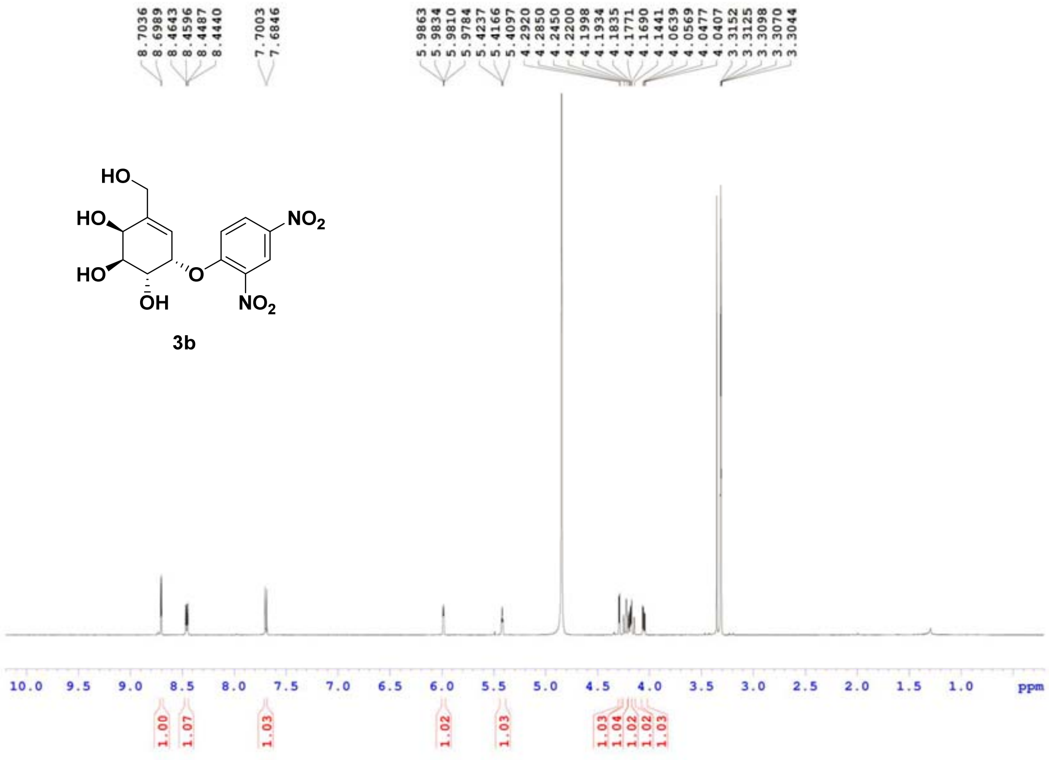

Supplementary Figure 20: ^{1}H NMR spectrum for **3b** in CD_3OD.

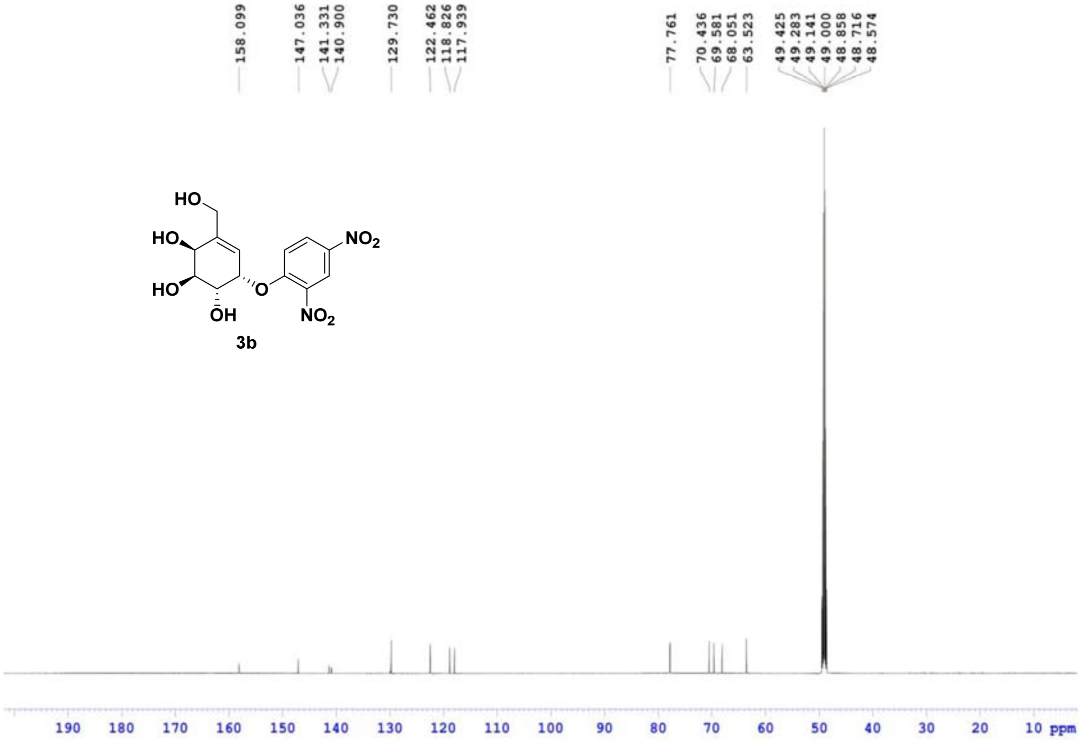

Supplementary Figure 21: ^{13}C NMR spectrum for **3b** in CD_3OD.

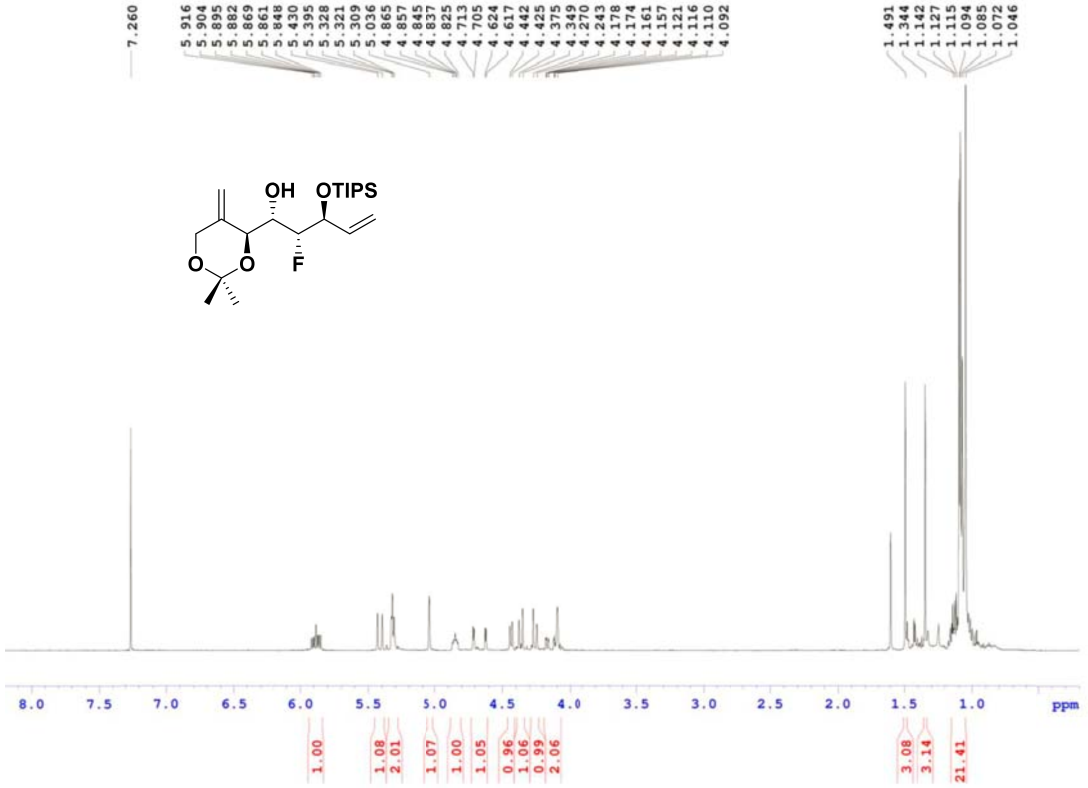

Supplementary Figure 22: ^{1}H NMR spectrum for **20** in $CDCl_3$.

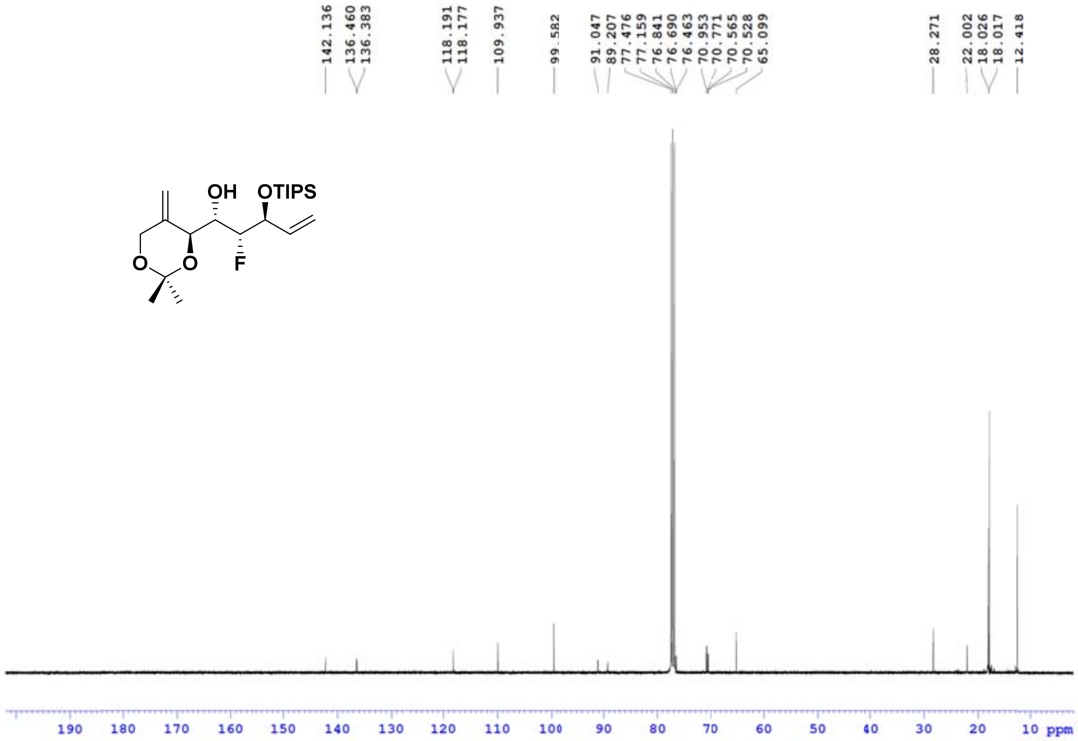

Supplementary Figure 23: ^{13}C NMR spectrum for **20** in $CDCl_3$.

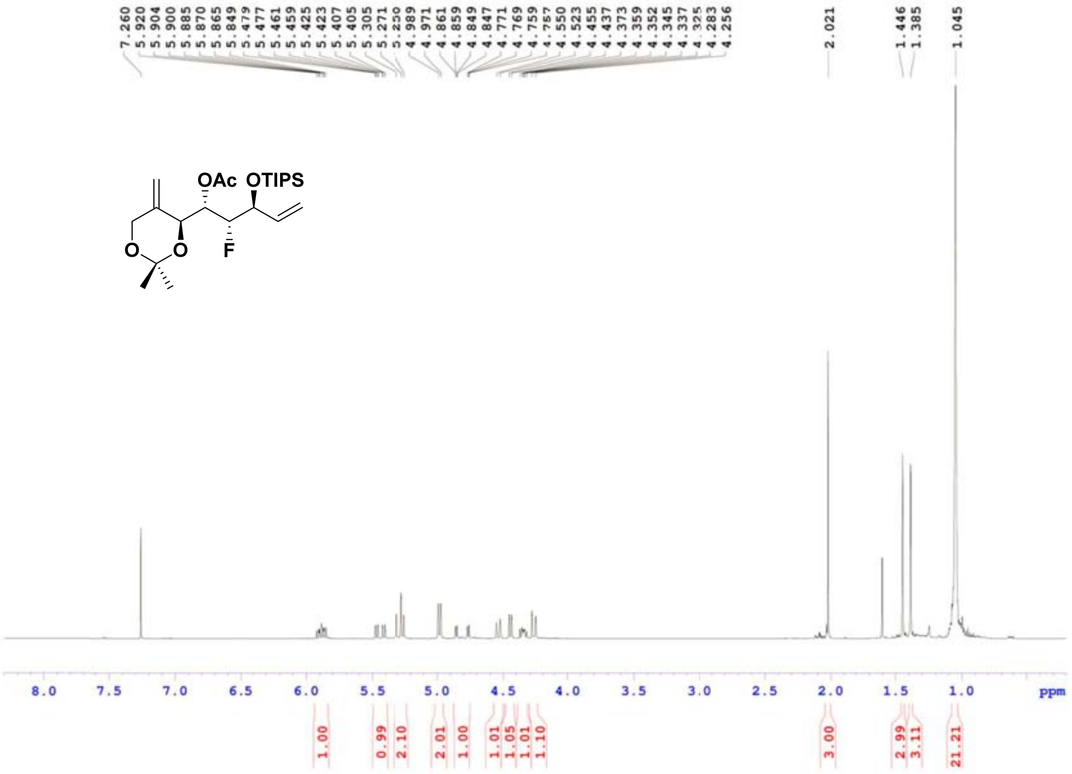

Supplementary Figure 24: ^{1}H NMR spectrum for **21** in $CDCl_3$.

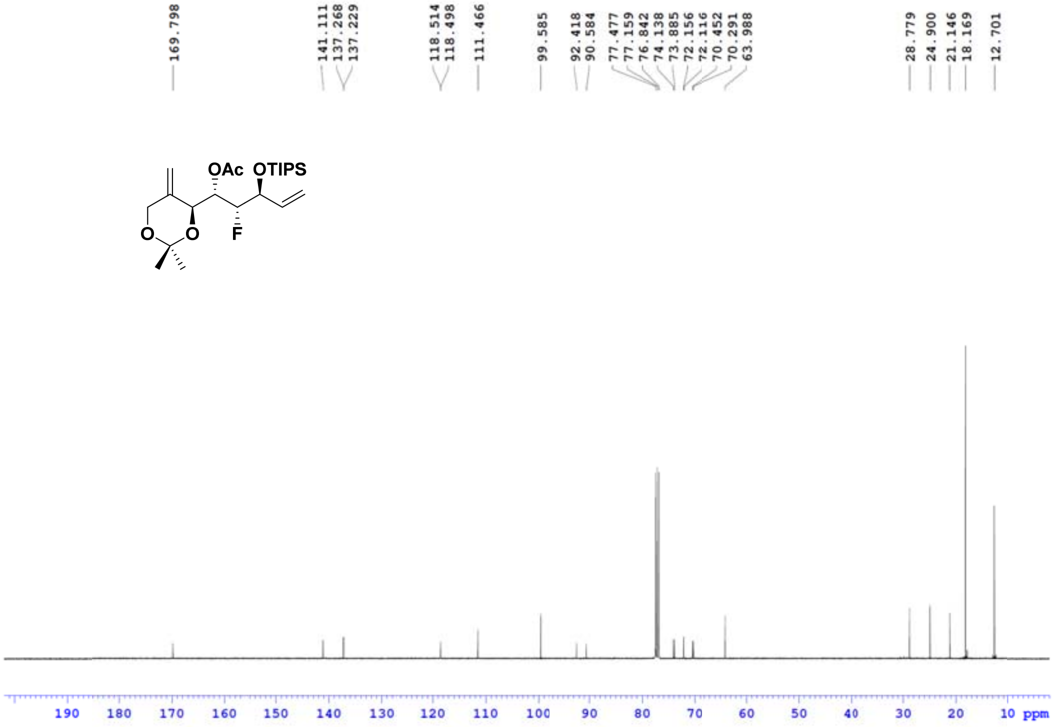

Supplementary Figure 25: ^{13}C NMR spectrum for **21** in $CDCl_3$.

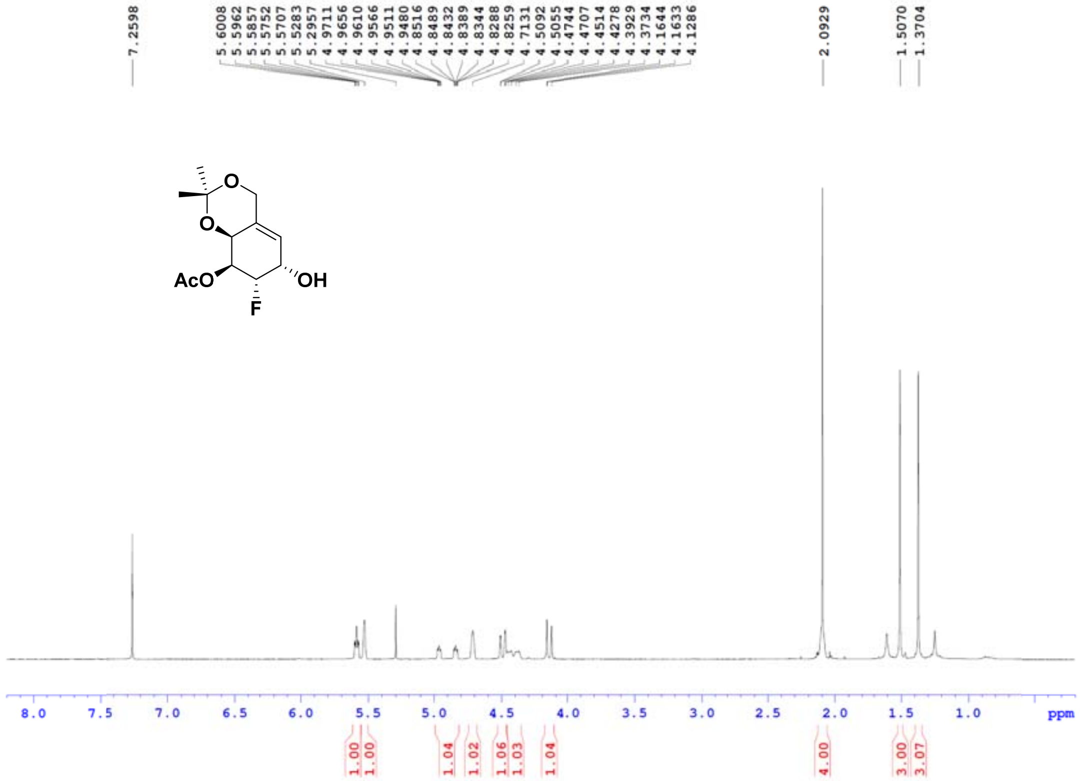

Supplementary Figure 26: ^{1}H NMR spectrum for **22** in $CDCl_3$.

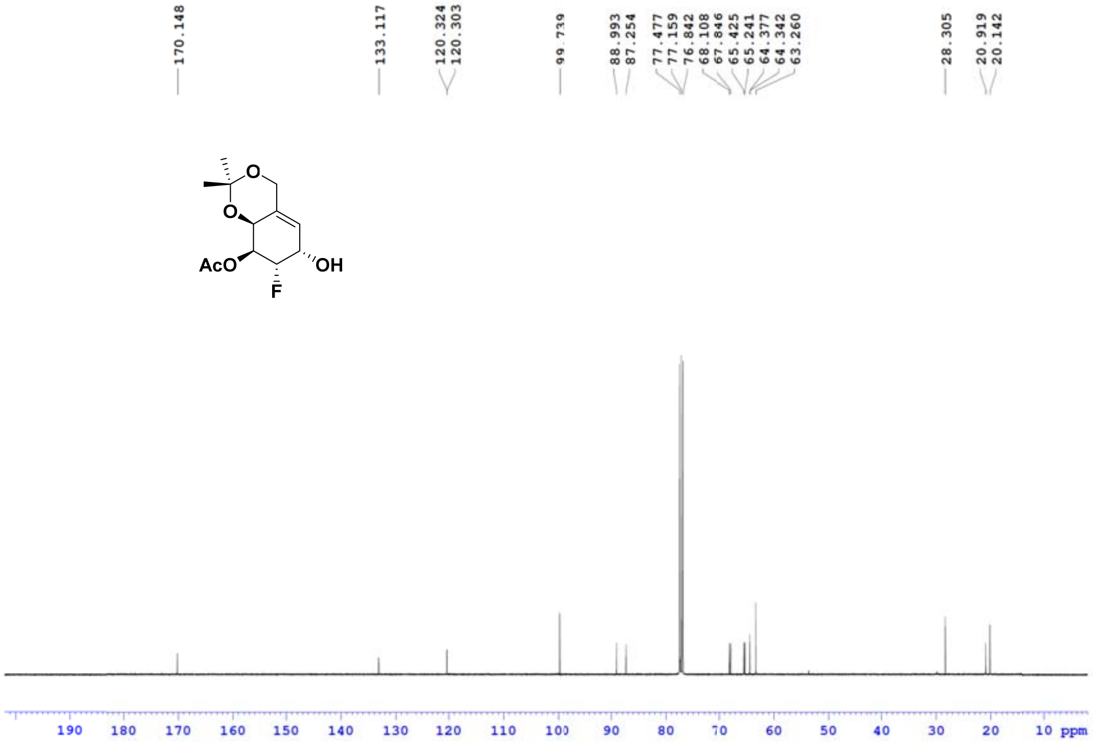

Supplementary Figure 27: ^{13}C NMR spectrum for **22** in $CDCl_3$.

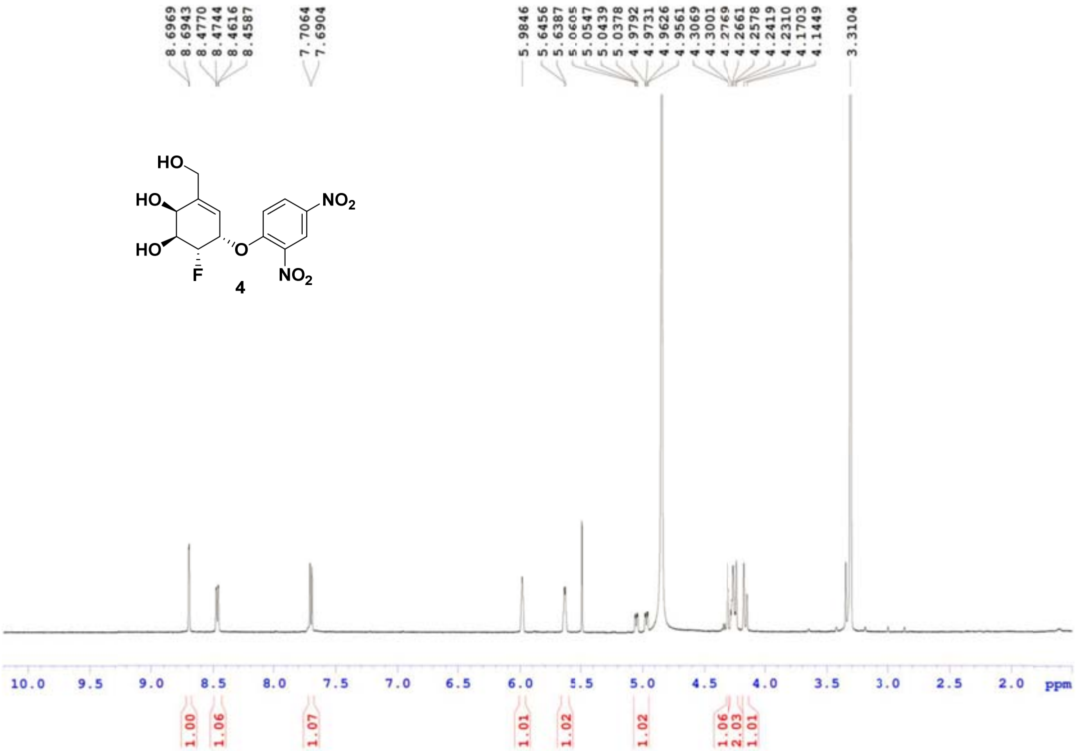

Supplementary Figure 28: ^{1}H NMR spectrum for **4** in CD_3OD.

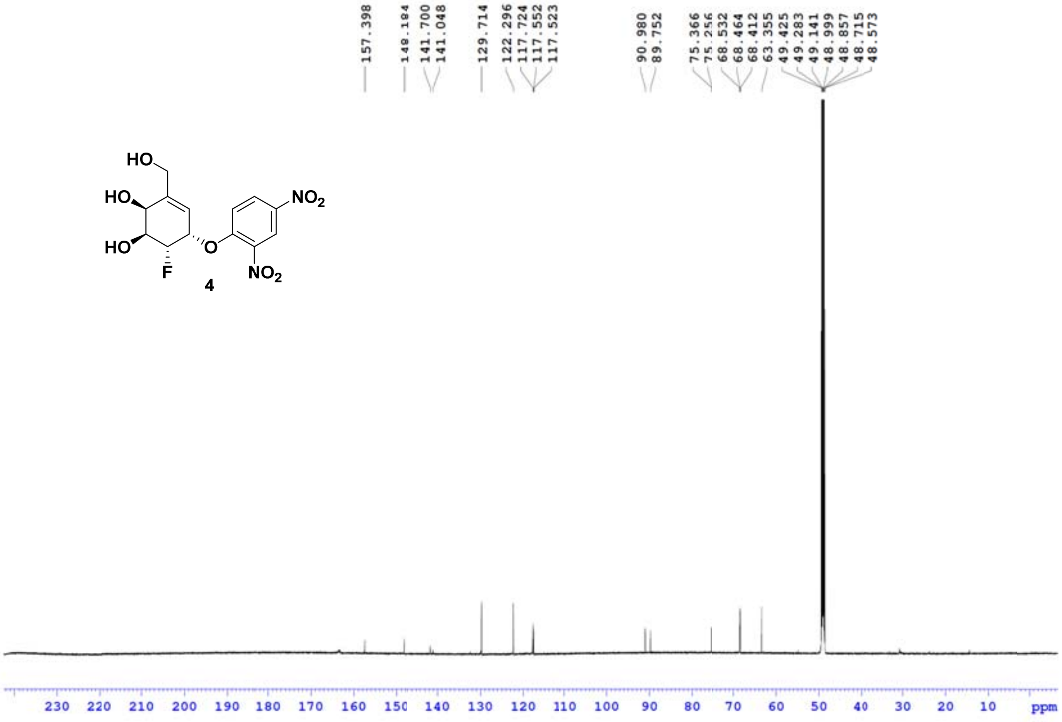

Supplementary Figure 29: ^{13}C NMR spectrum for **4** in CD_3OD.

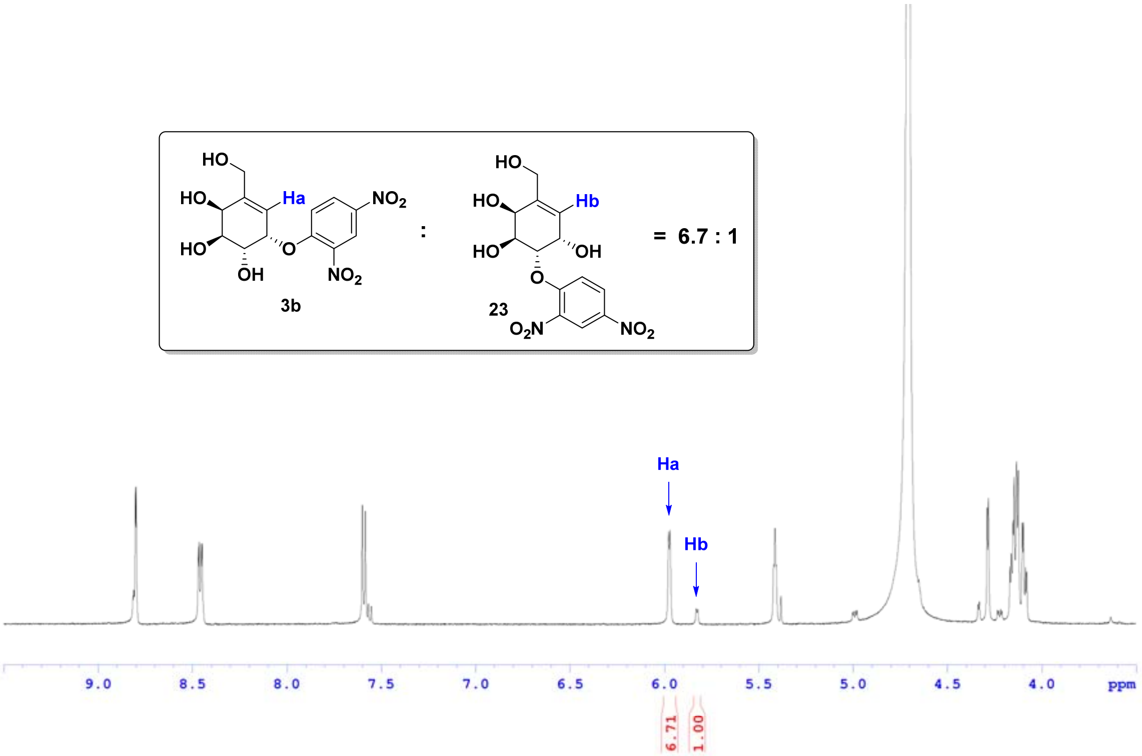

Supplementary Figure 30: Initial ^{1}H NMR spectrum for equilibration of compounds **3** and **23** (in D_2O at rt)

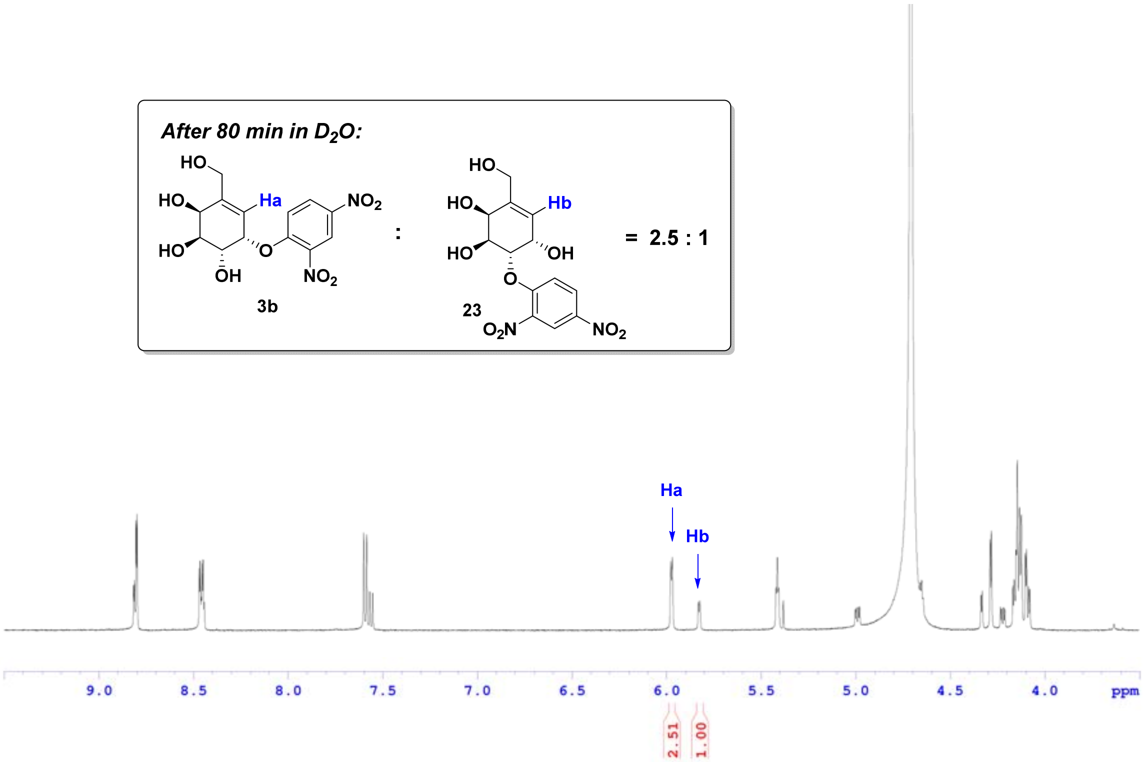

Supplementary Figure 31: ^{1}H NMR spectrum for the equilibration of **3** and **23** (in D_2O at rt) after 3 hrs.

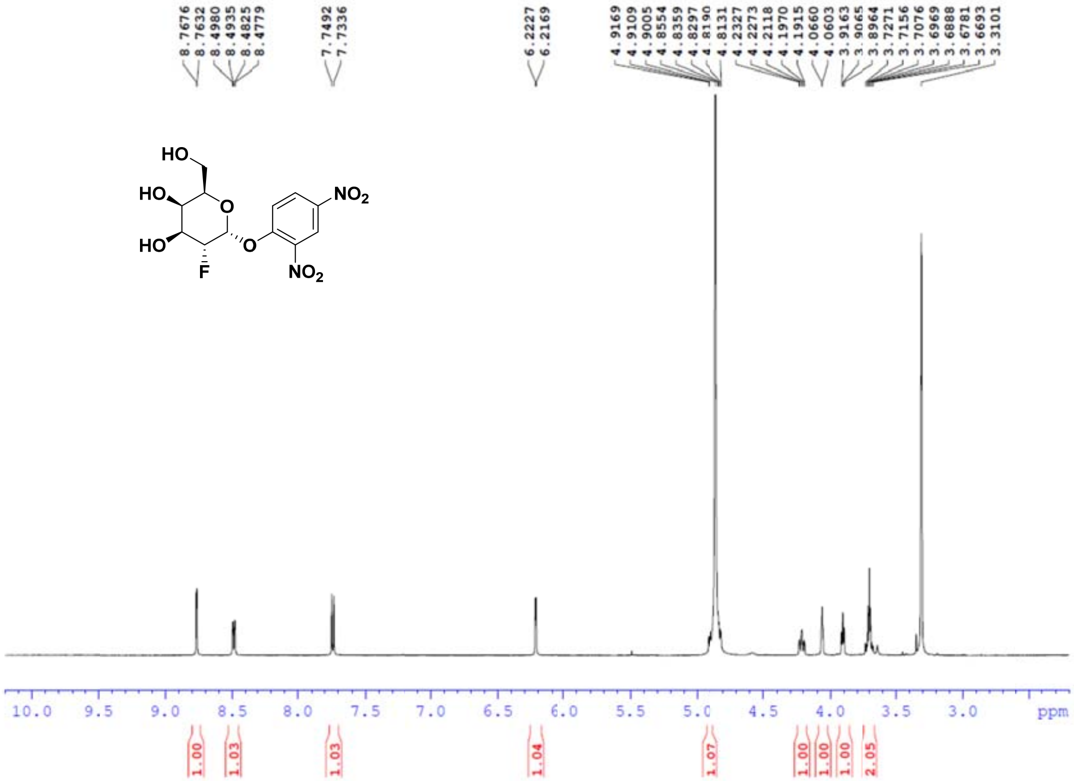

Supplementary Figure 32: ^{1}H NMR spectrum for **7** in CD_3OD.

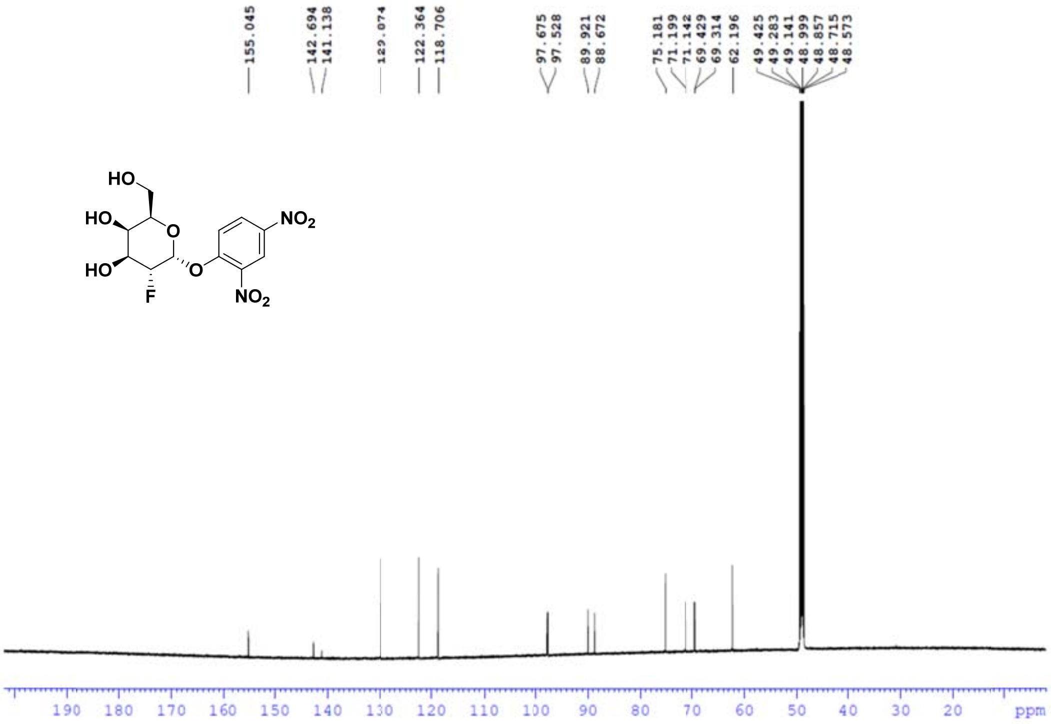

//

Supplementary Figure 33: ^{13}C NMR spectrum for **7** in CD_3OD.

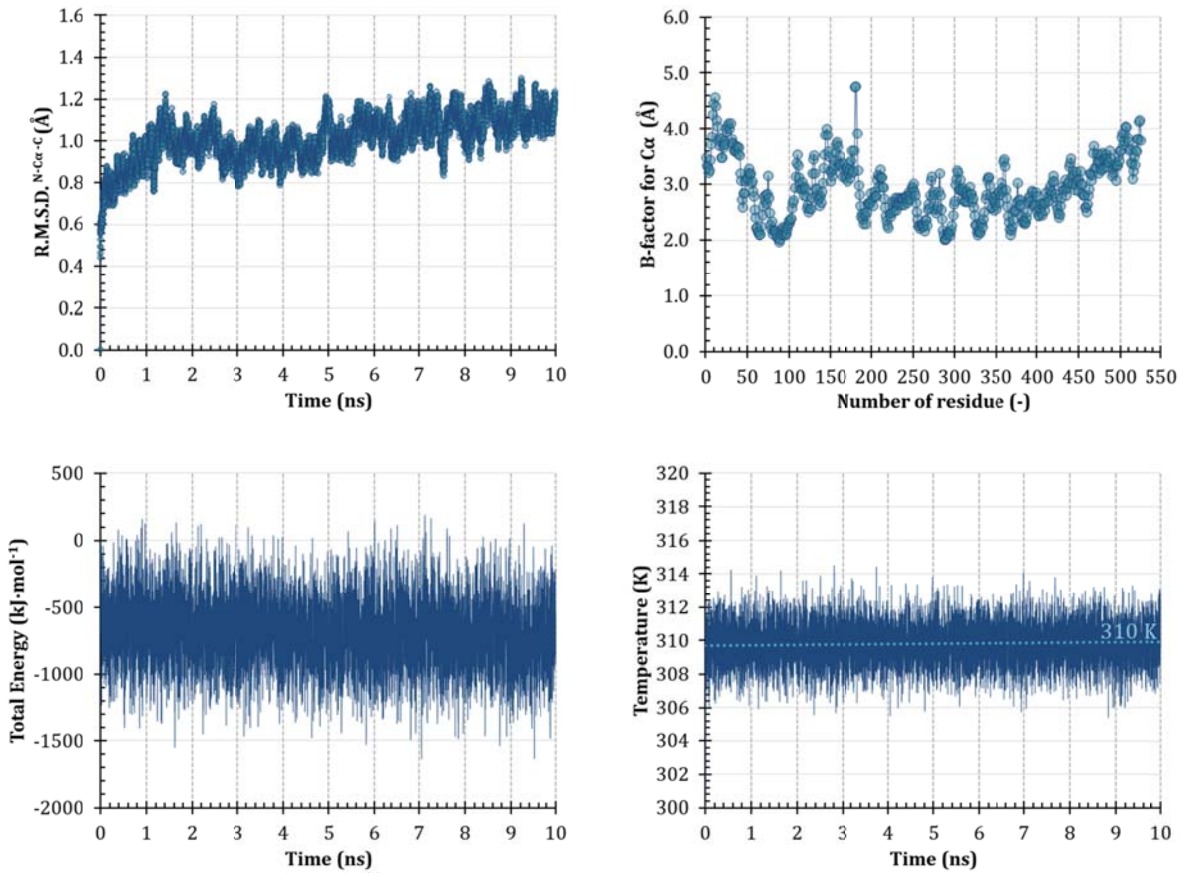

Supplementary Figure 34. Time dependence of RMSD, B-factor for C-α atoms, Total Energy and Temperature during 10 ns MM MD simulations performed to equilibrate the starting structure generated from the X-ray structure.

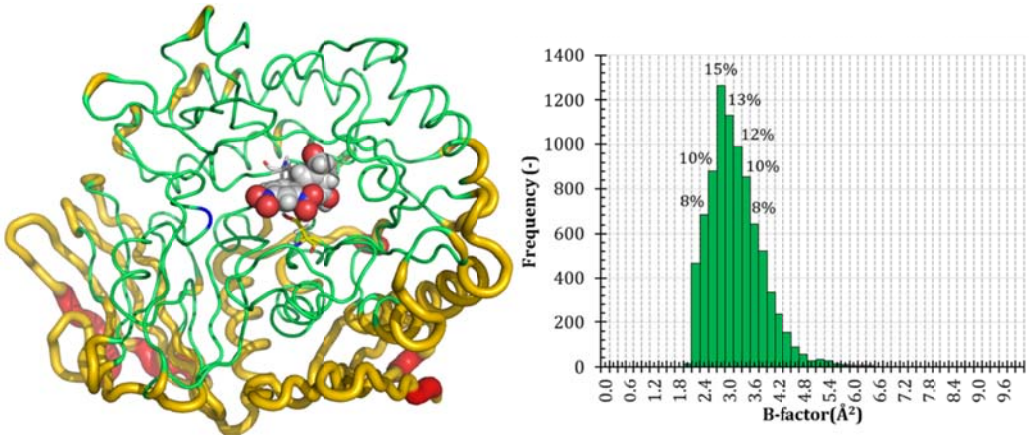

Supplementary Figure 35. Graphical representation of the B-factor along the protein backbone (left panel) and population analysis by residue (right panel).

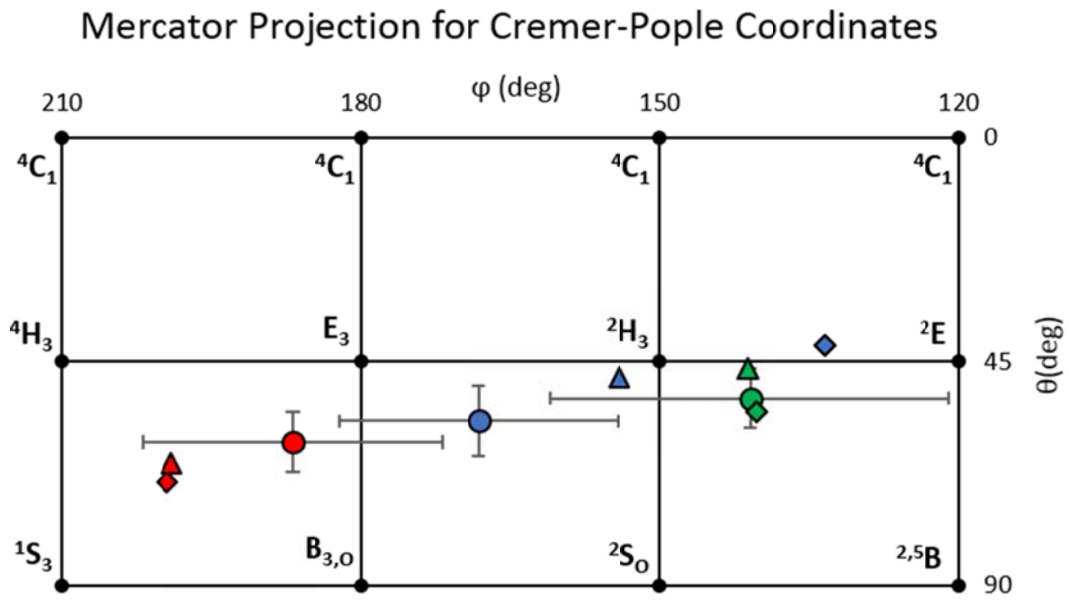

Supplementary Figure 36. Mercator projection representation of Cremer-Pople puckering coordinates for the structures generated from the M06-2X/MM (triangles) and AM1/MM (circles) calculations, and from the X-ray structures (diamonds) corresponding to the **E:I** (green symbols), **E-I** (red symbols) and **E:P** (blue symbols). Values obtained from data listed in Supplementary Table 4. The error bars represent standard deviations from the arithmetic mean of the puckering coordinates measured for 10 AM1/MM structures obtained from the 100 ps MD simulations in each conformation.

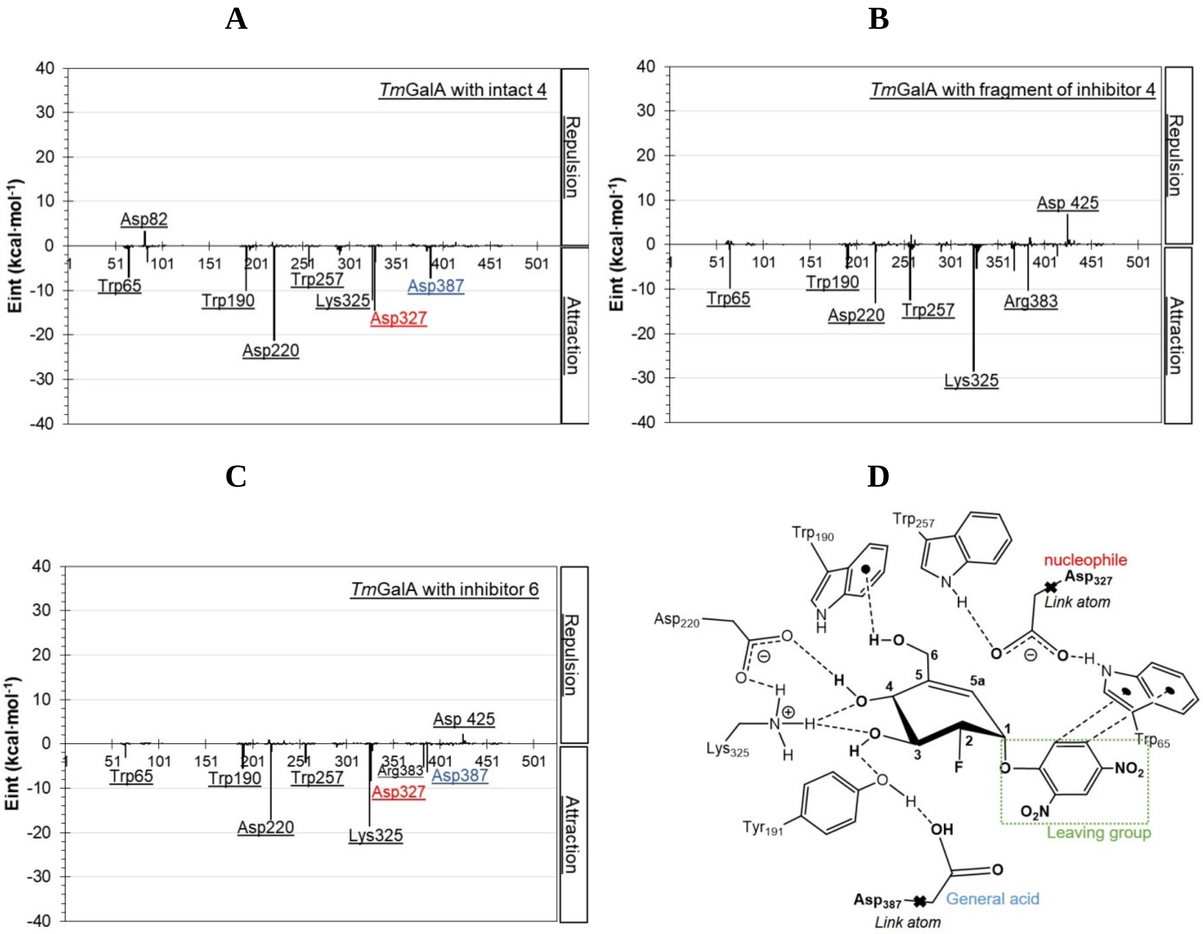

Supplementary Figure 37. Contributions of individual aminoacid residues to inhibitor interaction energy (in kcal/mol) averaged over 1000 structures generated along the AM1/MM MD simulations initiated from optimized structures for the different *Tm*GalA structures: **A** intact **4**; **B** 2-deoxy-2-fluorocarbagalactose fragment of **4** covalently bound to the nucleophile Asp327; and **C** inhibitor **6.** A schematic representation of the active site in the **E:I** complex is displayed in panel **D.**

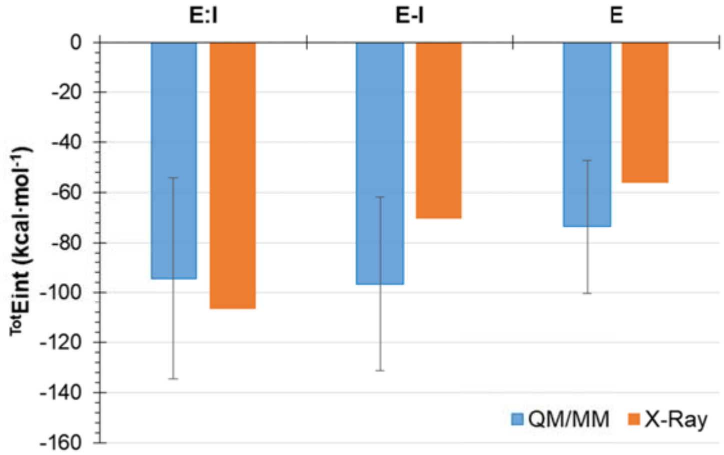

Supplementary Figure 38. Graphical representation of the total energy of interaction between the substrate and protein in the three key states (**E:I**, **E-I** and **E:P**) derived from the QM/MM structures (in blue) and the X-ray structures (in orange). Standard deviations on the structures derived from the QM/MM MD simulations are represented as bars.

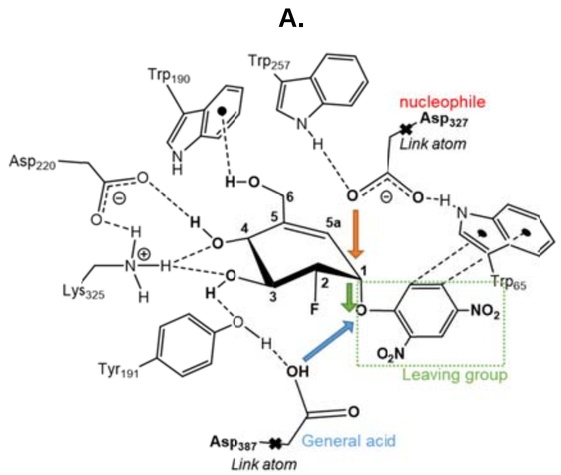

Supplementary Figure 39. Schematic representation of the active site of GH used for: A) the localization and optimization of covalent intermediate formation, **E-I**; and B) the hydrolysis of covalently-bound inhibitor to give **E:P**. The side chain of both Asp327 and Asp387, together with full inhibitor (A) or the remaining part of inhibitor and one water molecule (B), were described at QM level.

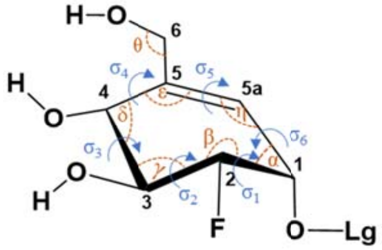

Supplementary Figure 40. Numbering and labeling of atoms and angles of the ring of inhibitor **4**.

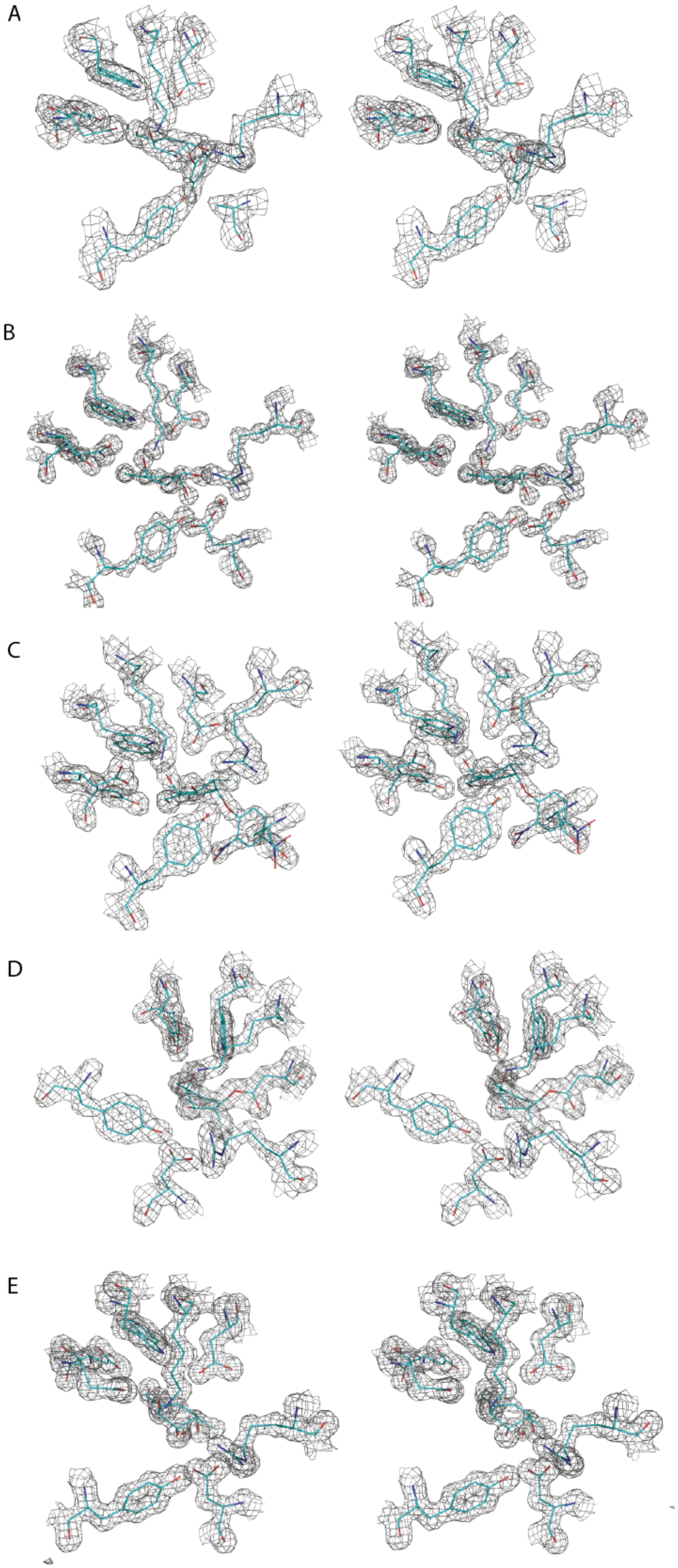
A
B
C
D
E

Supplementary Figure 41. Divergent stereo images to illustrate electron density maps for compounds **3a**, **4**, **5** and **6** with active site residues. (A) *Tm*GalA in complex with **3a**, (B) *Tm*GalA in complex with **5**, (C) *Tm*GalA in complex with **4**, (D) *Tm*GalA in complex with fragment of **4** covalently linked to D327, (E) *Tm*GalA in complex with **6**. In each case the maximum likelihood/σ_A weighted $2F_{obs}$ - F_{calc} electron denisty map is shown with contouring at 1.5 sigma in (A), 3 sigma in (B), 2 sigma in (C), 2 sigma in (D) and 1.8 sigma in (E).

Supplementary Table 1 Data collection and refinement statistics for *Tm*GalA in complex with 3a and 5.

	*Tm*GalA-D387A in complex with **3a** (6GTA)	*Tm*GalA in complex with **5** (6GVD)
**Data collection**		
Space group	$P2_12_12_1$	$P2_12_12_1$
Cell dimensions		
a, *b*, *c* (Å)	60.9, 97.9, 98.7	67.0, 96.1, 97.9
α, β, γ (°)	90.0, 90.0, 90.0	90.0, 90.0, 90.0
Resolution (Å)	68.50-2.20 (2.27-2.20) *	68.59-1.22 (1.25-1.22)
R_{merge}	0.104 (0.737)	0.064 (0.802)
R_{pim}	0.065 (0.518)	0.029 (0.506)
$I / \sigma I$	13.2 (2.0)	10.8 (2.2)
Completeness (%)	99.8 (99.5)	98.8 (93.5)
Redundancy	6.3 (5.4)	5.7 (3.9)
$CC_{1/2}$	0.998 (0.737)	0.996 (0.497)
**Refinement**		
Resolution (Å)	68.50-2.20	68.59-1.22
No. reflections	29040	173911
R_{work} / R_{free}	17.1 / 23.7	14.9 / 18.8
No. atoms		
Protein	4304	4511
Ligand/ion	37	25
Water	213	810
B-factors		
Protein	37.4	14.7
Ligand/ion	47.4	14.0
Water	38.0	33.5
Overall	37.5	17.6
R.m.s. deviations		
Bond lengths (Å)	0.007	0.008
Bond angles (°)	1.09	1.29

*Values in parentheses are for highest-resolution shell.

Supplementary Table 2 Data collection and refinement statistics for *Tm*GalA in complex with 4 and 6.

	*Tm*GalA-D387A in complex with **4** (6GWF)	*Tm*GalA in covalent complex with fragment of **4** (6GWG)	*Tm*GalA in complex with **6** (6GX8)
**Data collection**			
Space group	$P2_12_12_1$	$P2_12_12_1$	$P2_12_12_1$
Cell dimensions			
a, *b*, *c* (Å)	67.3, 95.9, 97.5	66.7, 95.9, 97.4	67.4, 95.8, 97.5
α, β, γ (°)	90.0, 90.0, 90.0	90.0, 90.0, 90.0	90.0, 90.0, 90.0
Resolution (Å)	67.32-1.72 (1.76-1.72) *	24.36-1.77 (1.82-1.77)	97.51-1.42 (1.46-1.42)
R_{merge}	0.145 (2.147)	0.118 (1.438)	0.055 (0.948)
R_{pim}	0.106 (2.615)	0.074 (0.903)	0.039 (0.742)
$I / \sigma I$	9.5 (2.5)	9.0 (1.1)	10.7 (1.5)
Completeness (%)	99.8 (99.5)	99.9 (56.4)	98.7 (93.9)
Redundancy	5.0 (4.9)	6.6 (6.7)	4.9 (3.6)
$CC_{1/2}$	0.997 (0.552)	0.997 (0.564)	0.999 (0.493)
**Refinement**			
Resolution (Å)	55.49-1.72	24.36-1.77	68.44-1.42
No. reflections	63960	58648	111449
R_{work} / R_{free}	16.5 / 20.3	16.7 / 21.5	17.4 / 20.2
No. atoms			
Protein	4395	4389	4428
Ligand/ion	35	66	34
Water	546	487	560
B-factors			
Protein	21.3	28.0	21.3
Ligand/ion	29.3	50.7	35.5
Water	35.5	38.3	35.9
Overall	22.9	29.4	23.1
R.m.s. deviations			
Bond lengths (Å)	0.010	0.009	0.010
Bond angles (°)	1.86	1.21	1.27

*Values in parentheses are for highest-resolution shell.

Supplementary Table 3. Missing atom types, charges and parameters for inhibitor **4**.

Atom name	Atom type	Charge (e^-)	**Missing parameters:**
C1	ca	-0.2021	IMPROPER
C2	ca	-0.0061	ca-ca-ca-ha 1.1 180.0
C3	ca	-0.2133	2.0
C4	ca	0.0079	ca-ca-ca-no 1.1 180.0
C5	ca	-0.2133	2.0
C6	ca	0.2200	ca-ca-ca-os 1.1 180.0
O1	os	-0.3030	2.0
C7	c3	0.1532	c2-c3-c2-ha 1.1 180.0
C8	c2	-0.1563	2.0
C9	c2	-0.1595	c2-c3-c2-c3 1.1 180.0
C10	c3	0.1645	2.0
C11	c3	0.1382	
O2	oh	-0.5909	
C12	c3	0.0950	
O3	oh	-0.5889	
C13	c3	0.1255	
F1	f	-0.2204	
O4	oh	-0.5689	
N1	no	0.3211	
O5	o	-0.1961	
O6	o	-0.1961	
N2	no	0.3281	
O7	o	-0.1926	
O8	o	-0.1926	
H1	ha	0.1779	
H2	ha	0.1849	
H3	ha	0.2059	
H4	h1	0.0776	
H5	ha	0.1599	
H6	h1	0.0676	
H7	h1	0.0677	
H8	ho	0.4110	
H9	h1	0.1067	
H10	ho	0.4030	
H11	ho	0.4150	
H12	h1	0.1127	
H13	h1	0.0567	

Supplementary Table 4. Puckering coordinates measured for the structures obtained from the QM/MM simulations and the X-ray structures and the final conformation label. a) Spherical coordinates (meridian angle φ, azimuthal angle θ, and radius Q) as defined in the Cremer-Pople; and b) the angles employed in the Hill-Reilly method.

Cremer-Pople puckering coordinates									
Param	**E:I**$^{X\text{-}Ray}$	**E:I**$^{AM1/MM}$	**E:I**$^{M06\text{-}2X/MM}$	**E-I**$^{X\text{-}Ray}$	**E-I**$^{AM1/MM}$	**E-I**$^{M06\text{-}2X/MM}$	**E:P**$^{X\text{-}Ray}$	**E:P**$^{AM1/MM}$	**E:P**$^{M06\text{-}2X/MM}$
ϕ (deg)	140.26	140.96 ± 20.08	141.15	199.43	186.90 ± 15.26	199.00	133.30	168.16 ± 14.30	153.95
θ(deg)	55.03	52.33 ± 5.61	46.42	69.34	61.08 ± 5.87	65.58	41.98	56.89 ± 6.74	48.09
Q (Å)	0.53	0.48 ± 0.03	0.49	0.56	0.52 ± 0.04	0.53	0.52	0.51 ± 0.06	0.51
**Conformer**:	^{2}E-$^{2}H_3$	^{2}E-$^{2}H_3$	^{2}E-$^{2}H_3$	E_3-$^{4}H_3$	E_3	E_3-$^{4}H_3$	^{2}E-$^{2}H_3$	$^{2}H_3$- E_3	$^{2}H_3$

Hill and Reilly angles of puckering									
Param	**E:I**$^{X\text{-}Ray}$	**E:I**$^{AM1/MM}$	**E:I**$^{M06\text{-}2X/MM}$	**E-I**$^{X\text{-}Ray}$	**E-I**$^{AM1/MM}$	**E-I**$^{M06\text{-}2X/MM}$	**E:P**$^{X\text{-}Ray}$	**E:P**$^{AM1/MM}$	**E:P**$^{M06\text{-}2X/MM}$
θ_0 (deg)	51.99	44.96 ± 3.79	48.20	20.28	27.58 ± 9.08	20.72	52.35	39.41 ± 10.79	46.83
θ_1 (deg)	10.79	12.05 ± 12.92	14.16	45.63	35.91 ± 9.27	42.96	13.40	25.29 ± 7.41	20.96
θ_2 (deg)	-11.51	-7.27 ± 8.05	-3.83	-34.16	-24.63 ± 5.34	-28.25	4.18	-20.08 ± 5.72	-9.72
**Conformer**:	$^{2}H_3$	$^{2}H_3$	$^{2}H_3$	E_3-$^{4}H_3$	E_3	$^{4}H_3$	^{2}E-$^{2}H_3$	$^{2}H_3$- E_3	$^{2}H_3$

Supplementary Table 5. Key intra-molecular distances (in Å) and angles (in degrees) of the stationary structures, Michaelis complex, **E:I**, Covalent Intermediate, **E-I,** and product complex of the hydrolysis, **E:P**, measured for the X-ray structures and those optimized at MM, AM1/MM and M06-2X/MM level of theory.

distance	E:I^{X-Ray}	E:I^{MM}	E:I$^{AM1/MM}$	E:I$^{M06-2X/MM}$	E-I^{X-Ray}	INT$^{AM1/MM}$	E-I$^{M06-2X/MM}$	E:P^{X-Ray}	E:P$^{AM1/MM}$	E:P$^{M06-2X/MM}$
d(C1-C2)	1.53	1.55±0.03	1.55 ± 0.03	1.52	1.58	1.55 ± 0.03	1.53	1.54	1.55 ± 0.03	1.52
d(C2-C3)	1.52	1.55±0.03	1.55 ± 0.03	1.52	1.48	1.55 ± 0.03	1.52	1.54	1.55 ± 0.03	1.52
d(C3-C4)	1.57	1.55±0.03	1.54 ± 0.03	1.53	1.58	1.54 ± 0.03	1.53	1.60	1.54 ± 0.03	1.53
d(C4-C5)	1.42	1.51±0.03	1.51 ± 0.03	1.52	1.41	1.51 ± 0.03	1.51	1.44	1.51 ± 0.03	1.52
d(C5-C5a)	1.39	1.33±0.02	1.34 ± 0.02	1.34	1.36	1.34 ± 0.02	1.33	1.36	1.34 ± 0.02	1.34
d(C5-C6)	1.46	1.51±0.03	1.50 ± 0.03	1.51	1.44	1.50 ± 0.03	1.50	1.45	1.50 ± 0.03	1.51
d(C5a-C1)	1.45	1.52±0.03	1.49 ± 0.03	1.50	1.45	1.49 ± 0.03	1.50	1.45	1.49 ± 0.02	1.50
d(C1-OLg)	1.50	1.45±0.03	1.47 ± 0.03	1.48	-	-	-	-	-	-
d(C1-OWAT)	-	-	-	-	-	-	-	1.50	1.43 ± 0.03	1.46
**angle**										
α(C5a-C1-C2)	112.1	112.7±2.6	112.1 ± 2.9	110.7	115.7	115.2 ± 2.6	115.9	107.6	113.5 ± 3.0	111.7
β(C1-C2-C3)	108.0	110.1±2.8	110.4 ± 2.9	111.0	112.0	112.3 ± 2.8	114.3	111.1	111.4 ± 3.3	110.9
γ(C2-C3-C4)	109.1	109.1±2.7	111.5 ± 3.1	110.6	108.8	111.0 ± 3.3	109.4	104.9	110.7 ± 3.3	109.2
δ(C3-C4-C5)	114.4	110.5±2.8	112.4 ± 3.0	111.6	111.3	110.3 ± 2.9	110.3	116.0	111.2 ± 2.8	110.8
ε(C4-C5-C5a)	121.6	122.2±2.4	123.2 ± 2.7	123.5	119.4	120.8 ± 2.8	120.0	120.6	122.2 ± 2.7	122.7
η(C5-C5a-C1)	123.6	124.1±2.5	124.1 ± 2.8	123.1	121.7	124.1 ± 2.7	122.2	125.5	124.2 ± 2.7	123.3
θ(C5-C6-OH)	116.0	109.2±3.5	112.3 ± 2.2	111.1	114.0	112.1 ± 3.2	112.3	121.9	112.4 ± 3.2	113.1
**dihedral**										
σ1(C5a-C1-C2-C3)	51.7	38.4±6.9	44.4 ± 6.7	50.7	5.3	17.1 ± 11.0	9.1	58.2	31.0 ± 14.1	46.5
σ2(C1-C2-C3-C4)	-63.2	-61.9±4.4	-57.4 ± 7.1	-62.3	-45.4	-49.0 ± 7.7	-46.8	-62.4	-55.4 ± 9.6	-64.5
σ3(C2-C3-C4-C5)	41.1	53.6±5.9	39.7 ± 10.0	42.3	62.5	54.8 ± 7.7	59.1	41.4	48.8 ± 8.1	48.8
σ4(C3-C4-C5-C5a)	-6.2	-23.8±6.8	-10.2 ± 9.9	-14.7	-34.0	-29.0 ± 8.5	-34.0	-17.9	-19.1 ± 9.8	-19.2
σ5(C4-C5-C5a-C1)	-6.3	0.6±6.7	-2.2 ± 8.6	4.9	-10.3	-4.2 ± 7.8	-6.0	13.5	-5.9 ± 8.4	2.4
σ6(C5-C5a-C1-C2)	-17.4	-8.1±8.1	-15.5 ± 8.7	-22.8	26.1	10.5 ± 11.1	19.2	-32.5	-0.4 ± 13.0	-15.9

Supplementary Table 6. Key inter-molecular distances (in Å) and angles (in degrees) of the stationary structures, Michaelis complex, **E:I**, Covalent Intermediate, **E-I,** and product complex of the hydrolysis, **E:P**, measured for the X-ray structures and those optimized at MM, AM1/MM and M06-2X/MM level.

distance	$E{:}I^{X\text{-}Ray}$	$E{:}I^{MM}$	$E{:}I^{AM1/MM}$	$E{:}I^{M06\text{-}2X/MM}$	$E\text{-}I^{X\text{-}Ray}$	$INT^{AM1/MM}$	$E\text{-}I^{M06\text{-}2X/MM}$	$E{:}P^{X\text{-}Ray}$	$E{:}P^{AM1/MM}$	$E{:}P^{M06\text{-}2X/MM}$
d(OAsp327-C1)	3.65	3.45±0.28	3.57 ± 0.33	3.22	1.40	1.46 ± 0.03	1.48	3.29	3.11 ± 0.19	2.87
d(C1-OLg)	1.50	1.45±0.03	1.47 ± 0.03	1.48	-	-	-	-	-	-
d(OAsp327- OLg)	5.12	3.45±0.28	5.00 ± 0.34	4.70	-	-	-	-	-	-
d(C1-OAsp387)	N.A	4.27±0.24	4.41 ± 0.24	4.33	5.00	6.38 ± 0.27	5.96	3.84	4.15 ± 0.28	4.60
d(C1-OWAT)	N.A	-	-	-	N.A	4.29 ± 0.39	3.46	1.50	1.43 ± 0.03	1.46
**angle**										
φ1(OAsp327-C1-OLg)	167.3	166.5±10.1	165.8 ± 6.1	174.3	N.A	-	-	N.A	-	-
φ2(OWAT-C1-OAsp327)	N.A	-	-	-	N.A	157.7 ± 5.4	168.3	168.3	143.0 ± 11.7	163.1

Supplementary Table 7. Key inter-atomic distances (in Å) measured between inhibitor **4** and important residues of GH in X-ray and in computationally obtained structures of Michaelis complex, **E:I**, Covalent Intermediate, **E-I,** and product complex of the hydrolysis, **E:P**, identified along the reaction pathway.

Distance	**E:I** $^{X\text{-}Ray}$	**E:I** $^{AM1/MM}$	**E-I** $^{X\text{-}Ray}$	**E-I** $^{AM1/MM}$	**E:P** $^{X\text{-}Ray}$	**E:P** $^{AM1/MM}$
F1-TRP65[NE1]	3.31	4.00±0.36	3.18	3.56±0.27	3.07	4.21±0.37
F1-CYS368[SG]	3.62	5.96±0.51	2.74	4.51±0.43	3.2	5.37±0.53
F1-ARG383[NH1]	3.07	5.38±0.37	3.29	5.28±0.38	3.07	4.49±0.30
F1-ARG383[NH2]	3.05	4.33±0.35	3.88	4.78±0.40	3.48	3.74±0.42
F1-ASP387[OD1]	NA	4.83±0.59	3.09	6.75±0.52	2.55	4.73±0.58
O4-TYR191[OH]	2.72	3.35±0.47	2.76	3.33±0.39	2.73	3.42±0.49
O4-LYS325[NZ]	2.73	3.07±0.26	2.89	3.10±0.25	2.83	2.97±0.18
O4-ARG383[NH1]	3.01	5.08±0.36	2.98	4.38±0.35	3.08	4.57±0.45
O3-ASP220[OD1]	2.69	2.73±0.13	3.32	2.74±0.13	2.67	2.77±0.14
O3-ASP220[OD2]	3.44	3.20±0.19	2.64	2.96±0.17	3.44	3.02±0.15
O3-TRP257[NE1]	3.13	3.72±0.50	2.95	3.64±0.52	3.19	3.75±0.45
O3-LYS325[NZ]	2.7	2.74±0.10	2.83	2.76±0.10	2.96	2.73±0.10
O2-ASP221[OD2]	2.68	4.91±0.27	2.73	5.34±0.29	2.63	5.70±0.32

Supplementary Table 8. Interaction energies by residue and total (E_{elec} + E_{VdW}) between inhibitor **4** and key residues of in X-ray and in computationally obtained structures of Michaelis complex **E:I**, covalent intermediate **E-I**, and product of hydrolysis **E:P**. Values given in kcal·mol^{-1}.

**Residue**	**E:I** $^{X\text{-}Ray}$	**E:I** $^{AM1/MM}$	**E-I** $^{X\text{-}Ray}$	**E-I** $^{AM1/MM}$	**E:P** $^{X\text{-}Ray}$	**E:P** $^{AM1/MM}$
Trp65	-5.75	-7.1 ± 0.2	-5.52	-9.8 ± 1.3	-2.84	-3.0 ± 0.9
Asp82	2.96	3.3 ± 0.5	-0.16	0.0 ± 0.1	-0.23	0.1 ± 0.1
Trp190	-15.6	-10.0 ± 1.6	-7.16	-5.3 ± 0.8	-8.37	-5.6 ± 0.9
Asp220	-20.22	-21.3 ± 3.3	-1.76	-13.1 ± 3.3	0.35	-17.2 ± 3.2
Trp257	-5.74	-4.6 ± 1.1	-8.87	-12.5 ± 1.5	-1.74	-4.2 ± 0.9
Lys325	-11.39	-12.3 ± 3.2	-15.95	-28.4 ± 3.1	-21.58	-18.6 ± 2.6
Asp327	-16.68	-14.5 ± 4.0	*bonded*	*bonded*	-16.91	-8.4 ± 2.1
Arg383	-9.19	-1.2 ± 1.5	-22.43	-10.4 ± 1.5	-8.54	-5.2 ± 1.4
Asp/Ala387	-4.28	-7.3 ± 1.8	3.97	-0.5 ± 1.5	-4.57	-6.3 ± 1.6
Asp425	2.83	-0.2 ± 0.6	6.25	6.8 ± 0.9	3.15	2.2 ± 0.8
**Total**	-106.5	**-94.4 ± 40.1**	-70.5	**-96.6 ± 34.7**	-56.3	**-73.6 ± 26.5**

Supplementary References.

1 Li, K. Y. *et al.* Synthesis of cyclophellitol, cyclophellitol aziridine, and their tagged derivatives. *Eur. J. Org. Chem.* 6030-6043 (2014).

2 Chakladar, S. *et al.* A mechanism-based inactivator of glycoside hydrolases involving formation of a transient non-classical carbocation. *Nat. Commun.* **5**, 5590 (2014).

3 Skaanderup, P. R., Poulsen, C. S., Hyldtoft, L., Jorgensen, M. R. & Madsen, R. Regioselective conversion of primary alcohols into iodides in unprotected methyl furanosides and pyranosides. *Synthesis* 1721-1727 (2002).

4 Namchuk, M. N., McCarter, J. D., Becalski, A., Andrews, T. & Withers, S. G. The role of sugar substituents in glycoside hydrolysis. *J. Am. Chem. Soc.* **122**, 1270-1277 (2000).
